# Quantifying the effect of organic aerosol aging and intermediate-volatility emissions on regional-scale aerosol pollution in China

**DOI:** 10.1038/srep28815

**Published:** 2016-06-28

**Authors:** Bin Zhao, Shuxiao Wang, Neil M. Donahue, Shantanu H. Jathar, Xiaofeng Huang, Wenjing Wu, Jiming Hao, Allen L. Robinson

**Affiliations:** 1 State Key Joint Laboratory of Environment Simulation and Pollution Control, School of Environment, Tsinghua University, Beijing 100084, China; 2 State Environmental Protection Key Laboratory of Sources and Control of Air Pollution Complex, Beijing 100084, China; 3 Center for Atmospheric Particle Studies, Carnegie Mellon University, 5000 Forbes Ave., Pittsburgh, PA 15213, USA; 4 Civil and Environmental Engineering, University of California, Davis, CA 95616, USA; 5 Key Laboratory for Urban Habitat Environmental Science and Technology, School of Environment and Energy, Peking University Shenzhen Graduate School, Shenzhen 518055, China

## Abstract

Secondary organic aerosol (SOA) is one of the least understood constituents of fine particles; current widely-used models cannot predict its loadings or oxidation state. Recent laboratory experiments demonstrated the importance of several new processes, including aging of SOA from traditional precursors, aging of primary organic aerosol (POA), and photo-oxidation of intermediate volatility organic compounds (IVOCs). However, evaluating the effect of these processes in the real atmosphere is challenging. Most models used in previous studies are over-simplified and some key reaction trajectories are not captured, and model parameters are usually phenomenological and lack experimental constraints. Here we comprehensively assess the effect of organic aerosol (OA) aging and intermediate-volatility emissions on regional-scale OA pollution with a state-of-the-art model framework and experimentally constrained parameters. We find that OA aging and intermediate-volatility emissions together increase OA and SOA concentrations in Eastern China by about 40% and a factor of 10, respectively, thereby improving model-measurement agreement significantly. POA and IVOCs both constitute over 40% of OA concentrations, and IVOCs constitute over half of SOA concentrations; this differs significantly from previous apportionment of SOA sources. This study facilitates an improved estimate of aerosol-induced climate and health impacts, and implies a shift from current fine-particle control policies.

Fine particles (i.e., particles with diameter of 2.5 μm or less (PM_2.5_)) have large impacts on human health^1^, and exert a significant but highly uncertain effect on climate forcing^2^. Organic aerosol (OA) accounts for 20–90% (30–70% in polluted atmospheres) of PM_2.5_ mass concentrations^3^,^4^, and 30–95% (30–80% in polluted atmospheres) of OA comprises secondary organic aerosol (SOA)^4^. SOA remains one of the least understood constituents of PM_2.5_; current widely-used models cannot predict the loadings or oxidation state of SOA^4^.

Recent studies have revealed that primary organic aerosol (POA), SOA, and the organic vapors in equilibrium with them together form a dynamic system that constantly evolves due to multi-generation oxidation^5^,^6^,^7^,^8^,^9^,^10^. First, POA, previously treated as nonvolatile and nonreactive, can evaporate, oxidize, and re-condense to form SOA, which is known as aging of POA^5^. Second, gas-phase oxidation products of traditional SOA precursors (i.e., non-methane volatile organic compounds, NMVOCs) can undergo multiple generations of oxidation, which has been demonstrated by smog-chamber experiments using gas-phase oxidation products as reactants^6^,^7^,^8^. Third, intermediate volatility organic compounds (IVOCs), currently not included or misclassified in emission inventories, have been shown to make a substantial contribution to the SOA budget in spite of being a small fraction of the overall organic gas emissions^9^,^10^,^11^. All of these new processes could lead to elevated SOA levels and oxidation state, but they are not accounted for in most chemical transport models (CTMs); inclusion can help explain the differences between model predictions and measurements.

Quantifying the effect of OA aging (including aging of POA and aging of SOA from traditional precursors) and intermediate-volatility emissions on the atmospheric OA budget and the OA oxidation state is challenging because it requires new frameworks to describe the continuous evolution of organic compounds. Some studies have simulated the aging of SOA from traditional precursors^12^,^13^,^14^,^15^,^16^,^17^,^18^,^19^ and/or the photo-oxidation of POA/IVOCs^5^,^13^,^14^,^16^,^17^,^18^,^20^,^21^,^22^ on a regional or global scale. Most of these studies have used a scheme based on lumping organic compounds into volatility bins, known as the volatility basis set (VBS, or 1D-VBS)^23^, which improved the model-measurement agreement compared with explicit chemical models (e.g., MCMv3.2^24^,^25^) and models based on empirical chamber fits (e.g., the two-product model). However, these studies have at least two drawbacks. First, the model frameworks are too simplified and some key reaction trajectories are not captured, such as the fragmentation process and the increase in OA oxidation state^4^. More importantly, the aging chemistry within these model frameworks is usually described phenomenologically based on analogous chemistry in smaller hydrocarbons. This is mainly because chamber experiments on OA aging and IVOC photo-oxidation were hardly available until recent years. Jathar *et al*.^26^,^27^ and Cappa *et al*.^28^ recently fitted the parameters of the statistical oxidation model (SOM) to chamber data to simulate the aging of SOA from traditional precursors, but the oxidation of POA and IVOCs was not considered.

Here we comprehensively assess the effect of OA aging and intermediate-volatility emissions on regional-scale OA pollution in China, which has high aerosol loadings^29^. We use a state-of-the-art model framework, the two-dimensional volatility basis set (2D-VBS)^6^,^30^, and incorporate it in a three-dimensional CTM for the first time. We constrain the model parameters using a series of SOA aging chamber experiments and POA/IVOC oxidation experiments, thereby achieving a more reliable assessment than previous studies. The findings of this study facilitate an improved estimate of aerosol-induced climate and health impacts, and imply a shift from current fine-particle control policies.

## Results and Discussion

### Simulation of SOA formation experiments

We simulated a series of SOA formation smog-chamber experiments with a 2D-VBS box model to determine the 2D-VBS parameters that agree best with measurements; there optimal parameters are subsequently used in three-dimensional CTMs. The 2D-VBS lumps the organic species into a space defined by volatility (effective saturation concentration, C^*^) and oxygen-to-carbon ratio (O:C), and describes the evolution of OA through two competing pathways: functionalization and fragmentation (see Supplementary Section 1). While most previous modelling frameworks track only organic mass, the 2D-VBS tracks O:C in addition to just organic mass, which allows us to impose a strong constraint on the model parameters by constraining the model predictions against both observed OA concentrations and O:C of OA.

In a previous paper^31^ we described simulation results for aging of SOA derived from typical traditional precursors, including toluene and α-pinene, which are anthropogenic and biogenic precursors with substantial emissions. We concluded that we should simulate the first-generation oxidation explicitly based on known chemistry and the subsequent aging chemistry with the 2D-VBS. With first-generation oxidation treated explicitly, we could find a group of 2D-VBS parameters that agrees well with the observations for toluene. Similarly, we could find another group of 2D-VBS parameters to achieve good model-measurement agreement for α-pinene. However, we are unable to simulate the oxidation of both toluene and α-pinene with the same 2D-VBS parameters.

Based on simulation results for toluene (10 experiments), α-pinene (6 experiments), pentadecane and C_13_ linear oxygenated precursors (6 experiments)^32^, we propose to use two parallel layers of the 2D-VBS (i.e., two different 2D-VBS configurations) in CTMs to simulate the aging of SOA derived from anthropogenic NMVOCs (AVOCs) and biogenic NMVOCs (BVOCs). This imposes little additional computational burden as in any event we separate anthropogenic and biogenic precursors to facilitate source attribution. We determined optimal parameters for each layer (Supplementary Table 1) by applying the least squares method to SOA concentrations and O:C across all experiments.

Evaporated POA and IVOCs are also important SOA precursors, so we also simulated a series of smog-chamber experiments using diluted emissions from major combustion sources^10^,^33^,^34^,^35^. The emission sources include gasoline vehicles (25 experiments), diesel vehicles (15 experiments), and biomass burning (18 experiments), which account for 77% of total POA emissions and 70% of combustion-related NMVOC emissions in China^36^,^37^,^38^. A key feature of these experiments is that individual emission sources in one class have distinct characteristics. For example, the gasoline vehicles spanned a range of model years, emission standards, vehicle types, mileage traveled, engine displacement, etc. The differences lead to large variability in precursor emission rates, precursor composition, and SOA production even for the same class of emission sources, as confirmed by Jathar *et al*.^10^. To simulate these experiments, we modeled the SOA formed from NMVOCs using SOA yields and modeled the photo-oxidation of POA and IVOCs with the 2D-VBS box model. The POA and IVOC emissions are distributed into the C^*^–O:C space based on distribution coefficients derived from measurements. In the base-case box-model simulation, the 2D-VBS parameters are exactly the same as our previous studies^6^,^31^ (see Methods for details of model configuration).

**Figure 1:**
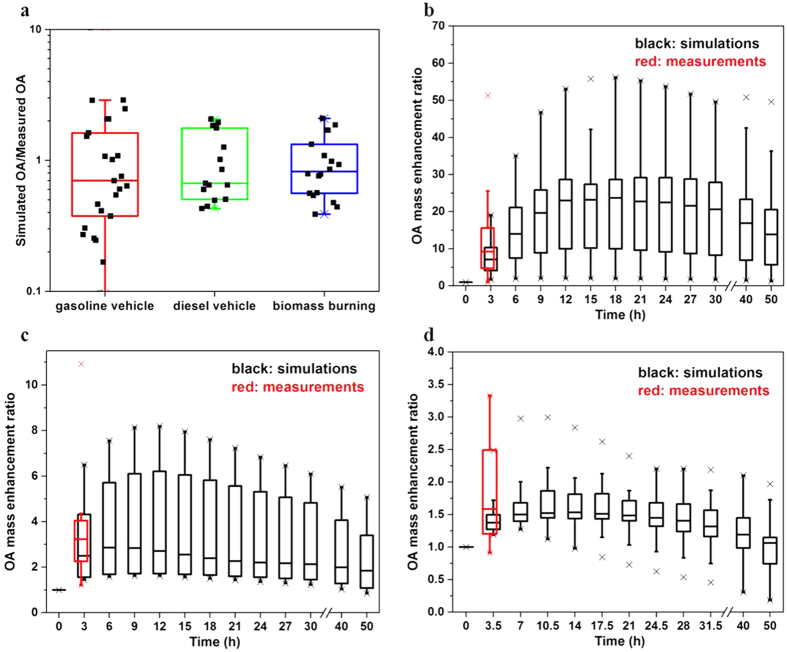
The base-case simulation results of smog-chamber experiments using diluted emissions from combustion sources. (**a**) The ratio of simulated to measured OA concentrations at the end of the experiments. Each black point represents an individual experiment. (**b–d**) The time-dependent simulated OA mass enhancement ratio for extended time for (**b**) gasoline vehicle experiments, (**c**) diesel vehicle experiments, and (**d**) biomass-burning experiments. The OA mass enhancement ratio is the ratio of the OA concentration at a specific time to the OA concentration at the beginning of an experiment. We also show the measured OA mass enhancement ratio at the end of the experiments (t = 3.0 h for gasoline/diesel vehicle experiments and t = 3.5 h for biomass-burning experiments). The OH concentrations in these experiments were 1.0–8.6 × 10^6^ molecule cm^−3^, comparable to typical atmospheric OH concentrations (about 3 × 10^6^ molecule cm^−3^). For all panels, the three horizontal lines of each “box” show the 25^th^ percentile (Q1), the median, and the 75^th^ percentile (Q3), respectively, and whiskers show the low and high extremes across the data. The low extreme extends to the data point closest to, but larger than Q1–1.5 × IQR, and the high extreme extends to the data point closest to, but smaller than Q3 + 1.5 × IQR, where IQR is the interquartile range, i.e., IQR = Q3–Q1. The symbols of “×” represent the maximum and minimum values.

Figure 1(a) compares predictions from base-case 2D-VBS parameters with measured OA concentrations at the end of the experiments. The simulation results are highly variable; the ratio of simulated to measured OA concentrations at the end of the experiments ranges between 0.2 and 3.0. This is likely due to differences in chemical composition of primary emissions in different experiments. For application in CTMs, the 2D-VBS mechanism describes the “average” behavior of organics, based on the assumption that every single C^*^–O:C bin comprises a diverse mixture of molecules^6^; therefore it does not capture differences in SOA yields due to different chemical composition within the bins. Furthermore, there is no evident principal factor (e.g., emission standard, vehicle type, mileage travelled, and so on) underlying the variability in SOA yields and model biases, probably because the characteristics of the emission sources are so different that the impact of any individual factor is obscured. Based on the median ratios of simulated to measured OA (0.67 to 0.82), the base-case 2D-VBS generally underestimates the measured OA concentrations at the end of the experiments.

The differences in model performance between the three source classes are relatively small compared with the large variability within each source class. Consequently, in CTMs, we propose to simulate the photo-oxidation of POA and IVOCs from all source classes with one additional unified layer of the 2D-VBS. In summary, we employ three parallel layers of the 2D-VBS with different configurations (Supplementary Table 1) in CTMs to simulate (1) aging of SOA from AVOCs, (2) aging of SOA from BVOCs, and (3) photo-oxidation of POA and IVOCs. Given the large model-measurement variability of the diluted emission experiments, we determined three sets of parameters (different from the base-case parameters) for the third layer of the 2D-VBS (Supplementary Table 1), “High-Yield VBS”, “Medium-Yield VBS” and “Low-Yield VBS” for which the 25^th^ percentile, the median value, and the 75^th^ percentile of the simulation to measurement ratios was 1.0, respectively (we show the simulation results in Supplementary Fig. 1).

Since the diluted emission experiments have limited OH exposure, we also predicted the evolution of the OA concentrations beyond the experiments at more atmospherically relevant OH exposure (Fig. 1(b–d)). For any of the three source classes, the model predicts further increases in OA concentrations beyond the experiments followed by a final decline as fragmentation reactions become dominant. The average peak OA concentrations account for about 22 times, 3 times, and 1.5 times those at the beginning of the experiments for gasoline vehicle, diesel vehicle, and biomass burning, respectively, with a large variability within each source class. It would be very useful to constrain the 2D-VBS parameters with experimental data under atmospherically relevant OH exposure. Unfortunately, experimental data at such OH exposure are very rare due to the limitation of commonly used smog-chambers. Recently, Tkacik *et al*.^39^ investigated the SOA formation from exhaust-dominated tunnel air (representing a mixture of all in-use vehicle emissions) using a potential aerosol mass (PAM) flow reactor, which allows much higher total OH exposure than smog-chambers. Tkacik *et al*.^39^ showed that, within 0.3–9.3 days of equivalent atmospheric oxidation, the peak OA concentrations account for about 4–11 times those at the beginning of the experiments, which is comparable to the OA enhancement ratio predicted by our 2D-VBS model. In future studies, more diluted emission experiments at atmospherically relevant OH exposure should be conducted and subsequently used to constrain the POA/IVOC oxidation parameters at longer photochemical ages.

### Simulation of OA and SOA in ambient air

Having developed the parameterization for the 2D-VBS, we incorporated it into the Community Multi-scale Air Quality model (CMAQ), a widely used CTM. We applied the CMAQ/2D-VBS model as well as the default CMAQv5.0.1 over a domain covering the vast majority of China (Supplementary Fig. 3). The CMAQv5.0.1 simulates NMVOC-derived SOA with a two-product model, treats POA as nonvolatile and nonreactive, and ignores IVOC emissions. In addition to NMVOCs and POA, the CMAQ/2D-VBS model requires emissions estimates for IVOCs. We quantified those emissions in the diluted emission experiments (last section) by extracting the speciated organic gases from the total non-methane organic gases (NMOG). On average, the IVOC emissions account for 30 times, 4.5 times, and 1.5 times the POA emissions from gasoline vehicles, diesel vehicles, and biomass burning, respectively. This factor was artificially set to 3.0 for other sources (industrial sources, coal-fired stoves) due to lack of measurements. We applied those factors to POA emissions to estimate IVOC emissions across the domain (see Methods for details of model development and configuration).

**Figure 2:**
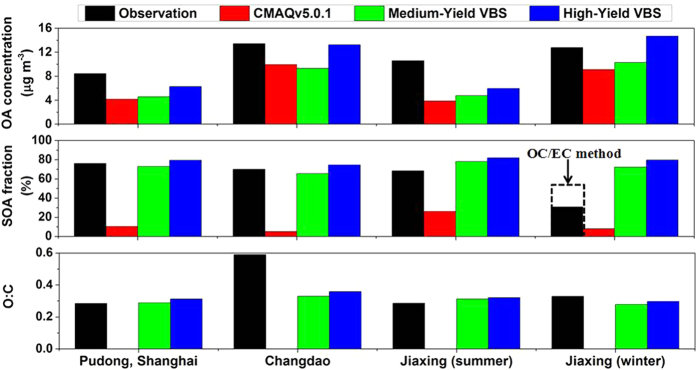
Comparison of simulated OA concentrations (top), fraction of OA consisting of SOA (middle), and O:C (bottom) with HR-ToF-AMS observations^49^,^50^,^51^. Simulation results of the Low-Yield VBS configuration are not shown because it results in quite low OA concentrations which are inconsistent with observations. The observed SOA corresponds to oxygenated organic aerosol (OOA) identified with the PMF technique based on HR-ToF-AMS data. The dashed rectangle in the middle panel represents the fraction of OA consisting of SOA obtained with the OC/EC method^49^.

We evaluated the simulation results using field observations obtained by both high-resolution time-of-flight aerosol mass spectrometer (HR-ToF-AMS; Fig. 2, Supplementary Table 4) and offline chemical analysis (Supplementary Figs 4–7, Supplementary Table 5). The observations are described in Supplementary Section 3.

The default CMAQv5.0.1 simulation significantly underestimates OA concentrations (OC for offline data) by 26% to 64% (45% on average). The Medium-Yield VBS simulation slightly improves the model performance, with an average OA underestimation of 40%. The High-Yield VBS simulation significantly improves the model performance, with a normalized mean bias (NMB) ranging between −44% and +15% (−19% on average). In addition, the Medium-Yield VBS and the High-Yield VBS show especially large improvement in model performance during heavy-pollution episodes (Supplementary Table 5 and Supplementary Figs 4–7).

More importantly, while the default CMAQv5.0.1 substantially underestimates the fraction of OA consisting of SOA by 5–10 times, the fraction of OA consisting of SOA simulated by the Medium-Yield VBS and the High-Yield VBS agrees well with observations for all sites and periods except for the Jiaxing site in winter (we discuss the reasons in Supplementary Section 3). Furthermore, while the aerosol O:C is not tracked in most models, the O:C in the Medium-Yield VBS and the High-Yield VBS agrees well with observations for all sites and periods (NMBs within ± 15%) except for the Changdao site (we discuss the reasons in Supplementary Section 3).

Based on the evaluation above, the High-Yield VBS configuration has the best performance. As described in the last section, the emission sources, e.g., gasoline vehicles, used in the diluted emission experiments are chosen to span a wide range of model years, emission standards, mileage traveled etc., rather than to represent the distribution of vehicles in current, in-use fleet. The Medium-Yield VBS configuration represents the median SOA yields of vehicles used in the experiments, but may not represent the average yield of the fleet in China. Therefore, the analysis in the next section is primarily based on the configuration that agrees best with field observations, i.e., the High-Yield VBS, with sensitivity analysis including the Medium-Yield VBS.

### Effect of OA aging and intermediate-volatility emissions

**Figure 3:**
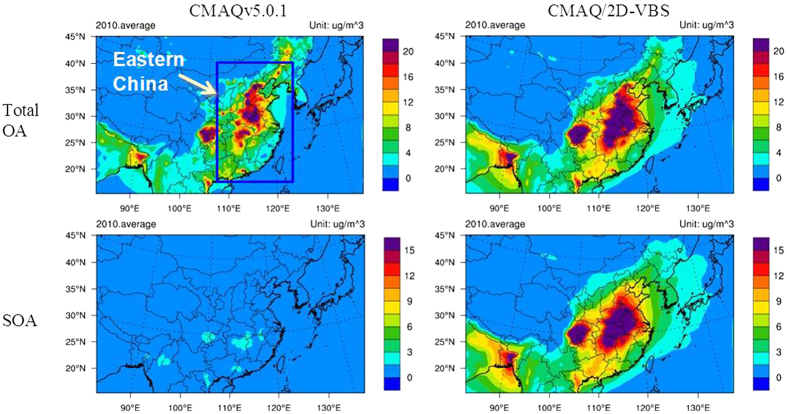
Spatial distribution of simulated 4-month (January, May, August, November) average OA and SOA concentrations in 2010. The left panels are for the default CMAQv5.0.1 and the right panels are for the High-Yield VBS. Substantial enhancements in OA and especially SOA are clearly evident in Eastern China for the High-Yield VBS compared with the default CMAQv5.0.1. The High-Yield VBS configuration predicts lower urban-to-regional OA concentration gradients than the default CMAQv5.0.1. Previous simulations using a 1D-VBS aging scheme have shown that these reduced urban-to-regional OA gradients agree better with observations in the Eastern US^5^. This figure is produced using the NCAR Command Language (Version 6.2.1) [Software]. (2014). Boulder, Colorado: UCAR/NCAR/CISL/TDD. http://dx.doi.org/10.5065/D6WD3XH5.

The differences in simulated OA and SOA concentrations between CMAQv5.0.1 and the High-Yield VBS (Fig. 3, Supplementary Figs 8 and 9, Supplementary Table 6) represent the effect of OA aging (including aging of SOA from traditional precursors and aging of POA) and intermediate-volatility emissions. It is clear that OA aging and intermediate-volatility emissions enhance OA concentrations in most seasons and regions (Fig. 3, Supplementary Figs 8 and 9); on average, they increase OA concentrations in Eastern China by 42% from 7.9 μg m^−3^ to 11.2 μg m^−3^. The increases are 30%, 64%, 56%, and 47% in January, May, August, and November, respectively (Supplementary Table 6). The larger fractional increase in summer is mainly attributable to higher solar fluxes and temperature accelerating photo-oxidation. For similar reasons, the increase is only 11% over the North China Plain, in contrast to 75%, 34%, and 43% over the Yangtze River Delta, the Pearl River Delta, and the Sichuan Basin, respectively (the spatial ranges of these regions are defined in Supplementary Fig. 3 and detailed statistics are given Supplementary Table 6). More importantly, the average SOA concentration in Eastern China is amplified dramatically by a factor of 10.6 from 0.8 μg m^−3^ to 8.6 μg m^−3^ with OA aging and intermediate-volatility emissions added. The increase is more than a factor of 6 for the average SOA concentration during any month and over any key region (Supplementary Table 6). In addition, Fig. 3 shows that the High-Yield VBS configuration predicts lower urban-to-regional OA concentration gradients than the default CMAQv5.0.1. Previous simulations using a 1D-VBS aging scheme have shown that these reduced urban-to-regional OA gradients agree better with observations in the Eastern US^5^.

The effect of OA aging can also be observed in the spatial and temporal variations of simulated O:C (Supplementary Fig. 10). The figure indicates that the simulated O:C in spring and summer is significantly higher than that of autumn and winter, which is consistent with a number of field observations^40^,^41^,^42^. A probable reason is that the photochemistry is more active in spring and summer due to stronger solar radiation and higher temperature. The aerosol O:C is the highest over the oceans, medium over Western China, and relatively low in Eastern China, because the regions with low emission rates are dominated by transport of aged OA.

**Figure 4:**
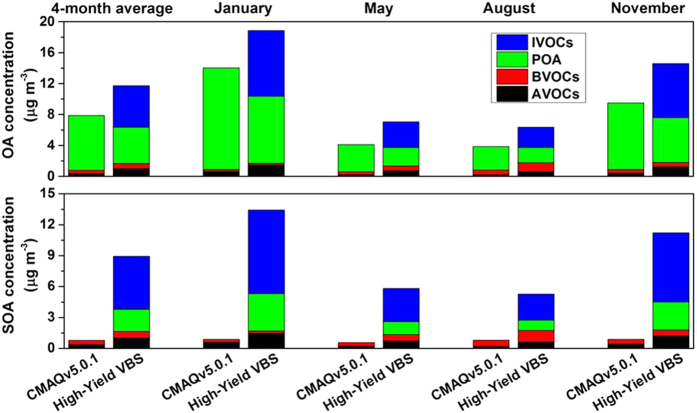
Contribution of individual precursor classes to OA and SOA concentrations in Eastern China. The contributions in key metropolitan regions are shown in Supplementary Table 7,8. The enhancements for the High-Yield VBS compared with CMAQv5.0.1 are especially pronounced for IVOCs (which are not treated in CMAQv5.0.1) and for POA (which has additional aging chemistry in CMAQ/2D-VBS).

Having assessed the overall effect of OA aging and intermediate-volatility emissions, we will evaluate the effect of individual processes, including aging of SOA derived from AVOCs and BVOCs, aging of POA, and photo-oxidation of IVOCs. The differences between the contributions of individual precursor classes (AVOCs, BVOCs, POA, and IVOCs) to OA concentrations derived from CMAQv5.0.1 and the High-Yield VBS (Fig. 4, Supplementary Table 7,8, Supplementary Section 4) represent the impact of these individual processes. Relative to the default CMAQv5.0.1, chemical aging increases SOA from AVOCs and BVOCs by 168% (141% to 208% in four seasons) and 54% (−22% toc +92%), respectively, in Eastern China (Fig. 4). In addition, Fig. 4 shows that the aging of POA decreases average OA concentration derived from POA by 33% from 7.1 μg m^−3^ to 4.7 μg m^−3^ in Eastern China. In other words, the decrease in OA concentrations owing to the evaporation of POA is not fully offset by gas-phase oxidation and re-condensation of the semi-volatile vapors. However, the aging of POA produces an average SOA concentration of 2.2 μg m^−3^, representing a new SOA source not treated in the default CMAQ. Finally, the oxidation of IVOCs is a substantial source of OA and SOA (5.3 μg m^−3^ on average in Eastern China, accounting for over half of total SOA loading), which is completely absent in the default CMAQ. This represents an integrated effect of adding IVOC emissions and its chemical aging. More studies are needed to separate the impacts of the two.

The simulation using the High-Yield VBS indicates that AVOCs, BVOCs, POA, and IVOCs contribute 9%, 5%, 40%, and 46% to average OA concentrations in Eastern China, respectively, and 11%, 7%, 24%, and 58% to average SOA concentrations, respectively. In spite of the seasonal and spatial variation (Supplementary Section 4), IVOCs and POA are the two largest contributors to OA concentrations, and IVOCs stand out as the largest contributor to SOA concentrations for most seasons and regions.

### Uncertainties and implications

We designed a series of sensitivity scenarios to assess the impact of key factors on simulated OA properties and the effect of OA aging and intermediate-volatility emissions (see Supplementary Section 5). Among these factors, the simulated OA concentrations are most sensitive to POA/IVOC emissions; the low POA/IVOCs and high POA/IVOCs scenarios can alter simulated OA concentrations by −35% and +109%, respectively. In almost all emission inventories, IVOC emissions are missing and POA emissions are quite uncertain, presenting an urgent need for further studies.

The uncertainty in POA/IVOCs oxidation chemistry also affects simulated OA concentrations significantly. Based on the Medium-Yield VBS, OA aging and intermediate-volatility emissions together increase average OA and SOA concentrations in Eastern China by 5% and a factor of 7.0, respectively (instead of 42% and a factor of 10.6 in the High-Yield VBS). As a result, simulated OA concentrations in the Medium-Yield VBS are about 28% lower than the High-Yield VBS. More studies should be conducted to reduce the large uncertainty in POA/IVOC oxidation chemistry.

In addition, we find that the simulated fraction of OA consisting of SOA is most sensitive to POA/IVOC oxidation chemistry and the volatility distribution of POA, and O:C distribution of fresh POA has the largest impact (~15%) on simulated aerosol O:C.

Despite the uncertainties, a conclusion from our simulations is that simulated SOA concentrations are dramatically increased by at least a factor of 7 in all these sensitivity scenarios as a result of OA aging and intermediate-volatility emissions; average OA concentrations are also increased in almost all sensitivity scenarios.

We should also note that the sensitivity scenarios above cannot fully account for the model-measurement discrepancies. For example, none of the uncertain parameters could individually account for the biases of O:C at the Changdao site and the SOA fraction at the Jiaxing site (winter). Some parameters may need to be varied together. Furthermore, the underestimation of OA concentrations even in the High-Yield VBS configuration may imply that some processes are still lacking or inadequately treated in the current model. A potentially influential process is the vapor wall loss in the chamber^43^. Cappa *et al*.^28^ showed that accounting for vapor wall losses leads to substantial increases in the simulated SOA concentrations from NMVOCs by factors of 2–5 and 5–10 for the low and high vapor wall-loss rate scenarios, respectively. Nevertheless, this should not alter our main conclusions that OA aging and intermediate-volatility emissions greatly increase SOA concentrations, and that IVOCs and POA make a substantial contribution to SOA concentrations. First, the fact that aging of SOA derived from NMVOCs increases SOA burden is independent of whether the chamber data are vapor wall-loss corrected or not. Second, even if we scale up SOA derived from NMVOCs by a factor of 5 in Fig. 4, the SOA formed from IVOCs and POA would still be at least comparable to that formed from NMVOCs. Beside vapor wall losses, the exclusion of aqueous SOA formation in the current model may also partly account for the remaining model-measurement discrepancies.

Underestimation of SOA is a major weakness in present understanding and model evaluation of aerosol-induced health effects and climate forcing. Furthermore, the oxidation state of OA is closely tied to hygroscopicity, and thus affects radiative forcing and cloud condensation nuclei formation^4^. Therefore, the improved simulation of OA/SOA concentrations and OA oxidation state in this study facilitates accurate estimation of the health damage and climate effect induced by aerosols.

The source apportionment of OA and SOA based on the CMAQ/2D-VBS differs significantly from those based on the default CMAQv5.0.1 and most previous CTMs. While the default CMAQv5.0.1 shows that POA is a dominant contributor to OA concentrations and NMVOCs are the exclusive precursors of SOA concentrations, the CMAQ/2D-VBS reveals that IVOCs and POA are the most important contributors to both OA and SOA concentrations over China. As discussed above, this conclusion should not be altered by the remaining uncertain factors, including those considered in the sensitivity scenarios, and the exclusion of vapor wall loss. Residential and commercial combustion and biomass open burning account for over 80% of total POA emissions in China. However, current control policies focus on large industrial sources and the control of these smaller sources does not receive enough attention. More importantly, NMVOC control policies may not be effective in reducing IVOC emissions; this problem has been noticed for the control technologies of gasoline vehicles in the United States^10^,^34^. The NMVOC control policies worldwide may need to be re-evaluated and significantly modified to ensure that IVOC emissions are effectively removed together with NMVOCs.

## Methods

### Simulation of smog-chamber experiments using diluted emissions from combustion sources

SOA precursors of these experiments include NMVOCs, POA, and IVOCs. Under the experimental conditions, SOA formed from NMVOCs undergoes limited multi-generation oxidation due to limited OH exposure, so we calculated this part of SOA formation using the SOA module of CMAQv5.0.1 and then removed that portion from SOA measurements in the smog-chamber data. We distributed POA and IVOC emissions in the C^*^–O:C space and simulated their multi-generation oxidation within the 2D-VBS framework. We determined the volatility distribution of POA emissions based on systematic measurements of May *et al*.^44^,^45^,^46^, who measured the same emission sources as we used in the 2D-VBS simulations. The measurements of the POA distribution in the O:C dimension are still quite limited. Aiken *et al*.^47^ measured the O:C of laboratory-produced highly-diluted POA emissions from gasoline/diesel vehicles and biomass burning to be 0.05 and 0.3–0.4, respectively, which reflected the O:C in the lower volatility range of POA. There have been more observations of OA in ambient air. Positive matrix factorization (PMF) of aerosol mass spectrometer (AMS) data can differentiate several types of OA, including hydrocarbon-like OA (HOA) and biomass-burning OA (BBOA), which assemble the POA emissions from fossil fuel combustion and biomass burning, respectively. We synthesized the AMS observations reviewed by Jimenez *et al*.^4^ around the world, by Ng *et al*.^48^ across the north hemisphere, and a series of observations in China^49^,^50^,^51^,^52^. The O:C of HOA and BBOA ranges between 0.04–0.16 and 0.19–0.27, respectively. Studies have also demonstrated a robust inverse correlation between O:C and volatility^48^,^53^,^54^. Based on the observational data above, we finalized the distribution coefficients of POA emissions in the C^*^ and O:C space (Supplementary Table 2). Then, for each experiment, we quantified the IVOC emissions as the difference between total NMOG emissions measured by flame ionization detection (FID) and speciated organic gases measured by gas chromatography–mass spectrometry (GC-MS), and distributed them into volatility bins of log_10_C^*^ = 4, 5, 6. Finally, the parameters in the base-case 2D-VBS to simulate multi-generation oxidation reactions are exactly the same as our previous studies^6^,^31^ and are described in Supplementary Section 1.

### Development and configuration of the CMAQ/2D-VBS model

We developed the CMAQ/2D-VBS model by incorporating a 2D-VBS box model into CMAQv5.0.1. The 2D-VBS introduced many new species, increasing the computational burden. We thus simplified the 2D-VBS to reduce runtime by about 70% while preserving very similar modeling results (see Supplementary Section 2).

We distributed the POA and IVOC emissions into the C^*^–O:C space. We determined the distribution parameters using the same method as the 2D-VBS box model (Supplementary Table 3). As for sources other than gasoline vehicles, diesel vehicles, and biomass burning (e.g., coal-fired stoves, industrial sources), we assumed their volatility distribution to be the average of the above three source classes, and their O:C to be the same as gasoline/diesel vehicles.

We simulated the first-generation reactions of traditional precursors explicitly based on known chemistry (mainly MCMv3.2^24^,^25^). We first modeled the formation of first-generation products for each precursor, and then derived the “average” first-generation products of each lumped species (e.g., “ARO1” for monoalkyl benzenes) based on the weighting factors of individual precursors constituting a lumped species, as assumed by Carlton *et al*.^55^ (see Supplementary Section 2 for details). Finally, we estimated the saturation concentrations of first-generation products using the Simplified P_L_^o^ (SIMPOL) prediction method^56^, and place them in the 2D-VBS.

We also added multi-generation oxidation reactions within the 2D-VBS framework, including (1) aging of SOA from AVOCs (2) aging of SOA from BVOCs, and (3) photo-oxidation of POA/IVOCs from all emission sources. Finally, we calculated the equilibrium gas-particle partitioning of organic compounds based on the total organic concentrations in all of the 2D-VBS layers.

We applied the CMAQ/2D-VBS model as well as the default CMAQv5.0.1 over a domain covering the vast majority of China and part of East Asia with a grid resolution of 36 km × 36 km (Supplementary Fig. 3). We used the default CMAQv5.0.1 as a reference to quantify the effect of OA aging and intermediate-volatility emissions. The default CMAQv5.0.1 was configured with the sixth-generation modal CMAQ aerosol model (AERO6). In AERO6, the thermodynamics of inorganic aerosols is simulated with ISORROPIA II^57^, and the NMVOC-derived SOA is estimated with a two-product model following Carton *et al*.^55^. AERO6 also includes a POA oxidation module developed by Simon *et al*.^58^, which we turned off in this study (i.e., assuming POA to be nonvolatile and nonreactive) to facilitate the quantification of aging effects. The chemical options of CMAQ/2D-VBS were the same as CMAQv5.0.1, except for modification of the SOA module as described above. We note that the smog-chamber data used to determine the 2D-VBS parameters for the aging of SOA from traditional precursors differ from those used to derive the yield parameters of CMAQv5.0.1. This may add to the uncertainty in quantifying the effect of aging, though Jathar *et al*.^26^ showed that the impact of different chamber data should be minor.

We used the Weather Research and Forecasting Model (WRF) version 3.3 to generate meteorological fields. The geographical projection, horizontal and vertical resolution, preparation of initial and boundary conditions, and physical options of WRFv3.3 were consistent with our previous paper^36^,^59^,^60^. The emission inventory is described in Supplementary Section 2.

The model simulation periods include January, May, August and November, 2010, representing the four seasons in 2010. We selected additional simulation periods based on availability of observational data. We defined five key metropolitan regions for region-specific analysis, as shown in Supplementary Fig. 3.

## Additional Information

**How to cite this article**: Zhao, B. *et al*. Quantifying the effect of organic aerosol aging and intermediate-volatility emissions on regional-scale aerosol pollution in China. *Sci. Rep.* **6**, 28815; doi: 10.1038/srep28815 (2016).

## Supplementary Material

Supplementary Information
