# Supplementary material for: Quantifying the effect of organic aerosol aging and intermediate-volatility emissions on regional-scale aerosol pollution in China
Source: Sci Rep. 2016 Jun 28;6:28815. doi: 10.1038/srep28815 (PMC4923863; doi:10.1038/srep28815)
Supplement: Supplementary Information [file srep28815-s1.doc]

# Supplementary material for the paper entitled:

Quantifying the effect of organic aerosol aging and intermediate-volatility emissions on regional-scale aerosol pollution in China

Bin Zhao^1, +^, Shuxiao Wang^1, 2, *^, Neil M. Donahue^3^, Shantanu H. Jathar^4^, Xiaofeng Huang^5^, Wenjing Wu^1^, Jiming Hao^1, 2^, and Allen L. Robinson^3^

^1^State Key Joint Laboratory of Environment Simulation and Pollution Control, Tsinghua University, School of Environment, Beijing 100084, China

^2^State Environmental Protection Key Laboratory of Sources and Control of Air Pollution Complex, Beijing 100084, China

^3^Center for Atmospheric Particle Studies, Carnegie Mellon University, 5000 Forbes Ave., Pittsburgh, PA 15213, USA

^4^Civil and Environmental Engineering, University of California, Davis, CA 95616, USA

^5^Key Laboratory for Urban Habitat Environmental Science and Technology, School of Environment and Energy, Peking University Shenzhen Graduate School, Shenzhen 518055, China

^+^Now at: Joint Institute for Regional Earth System Science and Engineering and Department of Atmospheric and Oceanic Sciences, University of California, Los Angeles, CA 90095, USA

Corresponding to: Shuxiao Wang (shxwang@tsinghua.edu.cn)

**1. Simulation of SOA formation experiments**

**2D-VBS framework and the base-case parameters.** The 2D-VBS framework is a powerful prognostic tool for modeling SOA evolution. It has been run either in a box-model mode[^1-3^](#_ENREF_1) or as a module of a Lagrangian transport model[^4-6^](#_ENREF_4) to simulate OA concentrations and oxidation state. In this study, the parameters of the base-case 2D-VBS to simulate the smog-chamber experiments using diluted emissions from combustion sources are exactly the same as for our previous studies. Below we will only review some key points of the model mechanism and parameters. The organic species are lumped into the C^*^ and O:C space with C^*^ ranging from 10^-5^ to 10^9^ μg m^-3^, separated by powers of 10, and O:C ranging from 0 to 1, separated by 0.1. The chemical mechanism in the 2D-VBS describes how material from one bin (a specific C^*^, O:C pair) is transformed after reaction, with OH radicals here, into products in a number of other bins. Here vapors react with OH at 3 × 10^-11^ cm^3^ molec^-1^ s^-1^, while heterogeneous OH reaction is 10 times slower because of gas-phase diffusion limitations to the particles. The 2D-VBS splits an oxidation process into two pathways: functionalization and fragmentation. Functionalization refers to reactions that add oxygenated functionality to the carbon backbone without breaking it. Fragmentation refers to reactions that break the carbon backbone. The functionalization scheme assumes that one generation of oxidation by OH results in products with C^*^ lowered by 1–6 (mostly 2–4) decades, and O:C increased via 1–3 added oxygen atoms. The probabilities for adding 1, 2, and 3 oxygen atoms are 30%, 50%, and 20%, respectively. The fragmentation scheme assumes that C-C bonds cleave at a random position along the carbon backbone (on average), generating fragments that are distributed between the C^*^ of the reactant and the most volatile C^*^ in the model; half of these fragments are radicals that immediately functionalize to form lower C^*^, more oxygenated stable products. The 2D-VBS framework tracks only stable molecule products rather than radical intermediates. Finally, we parameterize the branching ratio (β) between functionalization and fragmentation (the ratio of fragmentation pathway in all reacted organic mass) with β = (O/C)^1/4^, reflecting the increasing chance to fragment with increasing oxygenation. Note that both the functionalization and fragmentation schemes neglect any NO_X_ dependency to the chemistry. What we describe above is the configuration of the base-case 2D-VBS, and the configurations for application in 3-D simulations are summarized in Supplementary Table 1.

**Supplementary Table 1.** Parameterization for the three parallel layers of 2D-VBS.

| Parameter^a^ | First layer – aging of SOA from AVOCs | Second layer – aging of SOA from BVOCs | Third layer – photo-oxidation of POA and IVOCs | | |
| --- | --- | --- | --- | --- | --- |
|  |  |  | Medium-Yield VBS | High-Yield VBS | Low-Yield VBS |
| Homogeneous rate constant for aging | 2.5 × 10^-11^  cm^3^ molec^-1^ s^-1^ | 2.5 × 10^-11^  cm^3^ molec^-1^ s^-1^ | 4 × 10^-11^ cm^3^ molec^-1^ s^-1^ | 5 × 10^-11^ cm^3^ molec^-1^ s^-1^ | 1.7 × 10^-11^ cm^3^ molec^-1^ s^-1^ |
| Branching ratio (β) | β=(O:C)^1/3^ | β=(O:C)^0.45^ | β=(O:C)^0.4^ | β=0.75× (O:C) | β=(O:C)^1/6^ |
| Range of the O:C dimension | [0, 2] | [0, 1] | [0, 1] | [0, 1] | [0, 1] |
| Probabilities for adding 1, 2, or 3 oxygen atoms per generation of oxidation | 20%, 40%, and 40% | 60%, 30%, and 10% | 30%, 50%, and 20% | 30%, 50%, and 20% | 30%, 50%, and 20% |

^a^ only the parameters that differ with 2D-VBS layers are shown.

**Supplementary Table 2.** The mass fraction of POA emissions and O:C in each volatility bin used in 2D-VBS box model simulation.

| log_10_C^*^ (μg m^-3^) | | -2 | -1 | 0 | 1 | 2 | 3 | 4 |
| --- | --- | --- | --- | --- | --- | --- | --- | --- |
| Gasoline vehicle | Mass fraction | 0.14 | 0.13 | 0.15 | 0.26 | 0.15 | 0.03 | 0.14 |
|  | O:C | 0.13 | 0.13 | 0.11 | 0.09 | 0.06 | 0.03 | 0 |
| Diesel vehicle | Mass fraction | 0 | 0.03 | 0.25 | 0.37 | 0.24 | 0.06 | 0.05 |
|  | O:C | 0.13 | 0.13 | 0.11 | 0.09 | 0.06 | 0.03 | 0 |
| Biomass burning | Mass fraction | 0.2 | 0 | 0.1 | 0.1 | 0.2 | 0.1 | 0.3 |
|  | O:C | 0.28 | 0.28 | 0.24 | 0.20 | 0.16 | 0.12 | 0.08 |

| **a** | **b** | **c** |
| --- | --- | --- |
|  | | |

**Supplementary Figure 1.** Simulation results of the smog-chamber experiments using diluted emissions from combustion sources under the three sets of parameters for three-dimensional chemical transport simulation. (**a**) average median value of the simulation to measurement ratios of the three source classes equals 1.0; (**b**) average 25^th^ percentile of the simulation to measurement ratios of the three source classes equals 1.0; (**c**) average 75^th^ percentile of the simulation to measurement ratios of the three source classes equals 1.0. The meanings of the symbols are the same as Fig. 1.

**2. Development and configuration of the CMAQ/2D-VBS model**

**Simplification of the 2D-VBS framework.** Incorporation of the 2D-VBS into a chemical transport model (CTM) introduces many new species. In order to reduce the computational burden, we simplified the 2D-VBS by reducing the number of bins in both the volatility dimension and the O:C dimension. The volatility dimension was simplified from 15 bins ([-5, 9] separated by 1) to 9 bins ([-2, 0, 1, 2, 3, 4, 5, 6, 7]). This simplification is justified because organic compounds in bins with log_10_C^*^ < -2 almost exclusively partition to the aerosol phase, and compounds in bins with log_10_C^*^ > 7 are explicitly represented in the NMVOC emission inventory. The O:C dimension was simplified from 21 bins ([0, 2] separated by 0.1) to 8 bins ([0, 0.1, 0.2, 0.4, 0.7, 1.0, 1.5, 2.0]) for the AVOCs layer, and from 11 bins ([0, 1] separated by 0.1) to 6 bins ([0, 0.1, 0.2, 0.4, 0.7, 1.0]) for the other two layers, respectively. Our numerical tests indicate that the simplification reduces the runtime by about 70%. In addition, the simulated OA concentration and O:C before and after simplification agree well with each other, as shown in Supplementary Fig. 2. We also keep the original version of CMAQ/2D-VBS without simplification, and users may choose to use or not use the simplified version.

|  | Before simplification | After simplification |
| --- | --- | --- |
| OA | 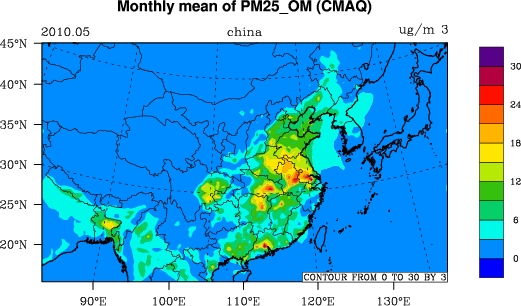 | 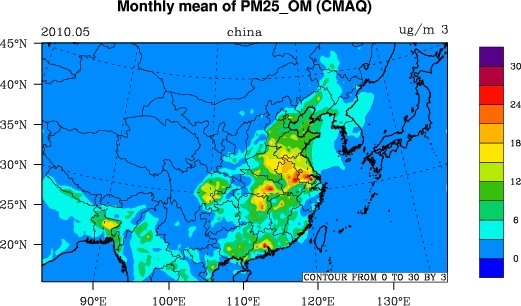 |
| O:C | 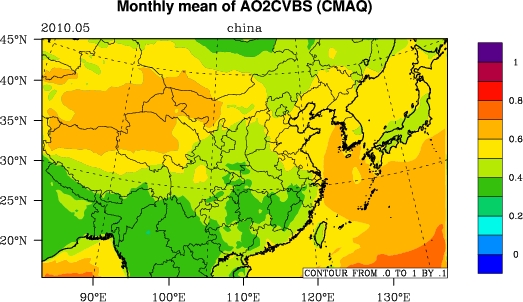 | 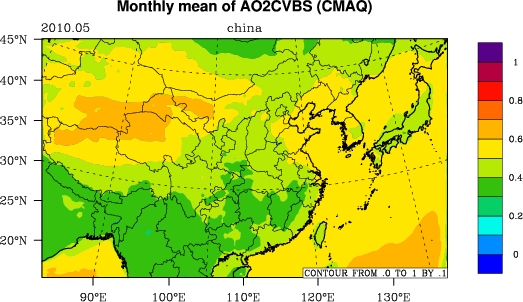 |

**Supplementary Figure 2.** Comparison of the CMAQ/2D-VBS simulation results before and after simplification. The simulation period is May 1^st^-5^th^. This figure is produced using the NCAR Command Language (Version 6.2.1) [Software]. (2014). Boulder, Colorado: UCAR/NCAR/CISL/TDD. http://dx.doi.org/10.5065/D6WD3XH5.

**Treatment of first-generation oxidation of traditional precursors.** The first-generation reactions of traditional precursors are simulated explicitly based on known chemistry. The traditional SOA precursors are categorized into 7 lumped species, including BENZ (benzene), ARO1 (monoalkyl benzenes), ARO2 (polyalkyl benzenes), ALK5 (long-chain alkane), ISOP (isoprene), TERP (monoterpene), and SESQ (sesquiterpene). We first modeled formation of first-generation products for each precursor, and then derived the “average” first-generation products of each lumped species based on the weighting factors of the precursors constituting a lumped species, as assumed by Carlton et al.[^8^](#_ENREF_8). The first-generation products and their distribution were obtained from MCMv3.2 (<http://mcm.leeds.ac.uk/MCMv3.2/>) for all precursors except for those lumped into ARO1, ARO2, and ALK5. For the precursors belonging to ARO1 and ARO2, we adopted the chemical species of the first-generation products from MCMv3.2, and the proportion of the bicyclic peroxy radical-dependent products from Ziemann and Atkinson[^9^](#_ENREF_9) (see Zhao et al.[^7^](#_ENREF_7) for details). As for ALK5, we have not treated the first-generation reactions explicitly but rather used the yield parameters of CMAQv5.0.1 to approximate the first-generation reactions, because the precursors lumped in ALK5 are numerous. Finally, we estimated the saturation concentrations of the first-generation products using the SIMPOL method[^10^](#_ENREF_10), and place them in the 2D-VBS.

**Development of emission inventory.** The anthropogenic emissions for East Asia were developed in our previous studies[^11-13^](#_ENREF_11) except for NH_3_ emissions from fertilizer application, which we calculated with the bi-directional CMAQ model coupled to an agro-ecosystem model[^14^](#_ENREF_14). We derived the emissions for other Asian countries from the INDEX-B inventory[^15^](#_ENREF_15). Since the majority of observational sites are located in the Yangtze River Delta (Supplementary Fig. 3), the largest city-cluster in China, we replaced the anthropogenic emissions in this region with a high-resolution local emission inventory[^16^](#_ENREF_16). The biogenic emissions were calculated by the Model of Emissions of Gases and Aerosols from Nature (MEGAN) version 2.04[^17^](#_ENREF_17).

**Supplementary Figure 3.** The CMAQ/2D-VBS modeling domain (black rectangle), the five key regions (blue rectangles), and the location of observational sites (colored stars). The Yangtze River Delta region is zoomed in to show the observational sites clearly. This figure is produced using DotSpatial, [version 1.7], (<http://dotspatial.codeplex.com/>) and Microsoft PowerPoint 2013 (https://www.microsoft.com/).

**Supplementary Table 3.** The mass fraction of POA emissions and O:C in each volatility bin used in CMAQ/2D-VBS simulation.

| log_10_C^*^ (μg m^-3^) | | -2 | 0 | 1 | 2 | 3 | 4 | 5 | 6 |
| --- | --- | --- | --- | --- | --- | --- | --- | --- | --- |
| Gasoline vehicle | Mass fraction^a^ | 0.27 | 0.15 | 0.26 | 0.15 | 0.03 | 6.14 | 10.5 | 13.5 |
|  | O:C | 0.13 | 0.11 | 0.09 | 0.06 | 0.03 | 0 | 0 | 0 |
| Diesel vehicle | Mass fraction | 0.03 | 0.25 | 0.37 | 0.24 | 0.06 | 0.95 | 1.575 | 2.025 |
|  | O:C | 0.13 | 0.11 | 0.09 | 0.06 | 0.03 | 0 | 0 | 0 |
| Biomass burning | Mass fraction | 0.2 | 0.1 | 0.1 | 0.2 | 0.1 | 0.6 | 0.525 | 0.675 |
|  | O:C | 0.28 | 0.24 | 0.20 | 0.16 | 0.12 | 0.08 | 0.08 | 0.08 |
| Other sources | Mass fraction | 0.167 | 0.167 | 0.243 | 0.197 | 0.063 | 0.763 | 1.05 | 1.35 |
|  | O:C | 0.13 | 0.11 | 0.09 | 0.06 | 0.03 | 0 | 0 | 0 |

^a^ the sum of mass fractions of all volatility bins exceeds 1.0 because both POA and IVOCs are included in this table.

**3. Evaluation of the CMAQ/2D-VBS simulation results**

**Description of the field observation data.** To evaluate the model performance, we collected a series of observational data in China. The HR-ToF-AMS could not only measure the major chemical components of aerosols (EC, OA, SO_4_^2-^, NO_3_^-^, NH_4_^+^), but also measure the elemental composition of OA and identify several types of OA with the positive matrix factorization (PMF) technique. These OA types include hydrocarbon-like OA (HOA), biomass-burning OA (BBOA), and oxygenated organic aerosol (OOA); OOA are sometimes further divided into semi-volatile OOA (SV-OOA) and low-volatility OOA (LV-OOA). Here HOA and BBOA are assumed to be POA, and OOA is assumed to be SOA. Therefore, HR-ToF-AMS provides high quality data for the evaluation of CMAQ/2D-VBS. The data we collected include (a) the observations at an urban site in Pudong, Shanghai by Huang et al.[^18^](#_ENREF_18) from May 15^th^ to June 10^th^, 2010; (b) the observations at a suburban site in Jiaxing by Huang et al.[^19^](#_ENREF_19) during June 29^th^ – July 16^th^ and December 10^th^ – December 24^th^, 2010; (c) the observations in Changdao island, a small county located in the Bohai Bay, by Hu et al.[^20^](#_ENREF_20) from March 21^st^ – April 24^th^.

However, HR-ToF-AMS observations are still quite rare across China, and usually last for short periods. Therefore, we collected additional observations of PM_2.5_ and its components (EC, OC, SO_4_^2-^, NO_3_^-^, NH_4_^+^) derived from offline sampling and chemical analysis by Tsinghua University. The sites are all located in the urban areas of the Yangtze River Delta, including two sites in Shanghai (Pudong and SAES), one in Nanjing, and one in Suzhou. The observation periods are May 20^th^ – June 30^th^, July 20^th^ – August 20^th^, November 7^th^ – 30^th^, and December 20^th^ – 31^st^, 2011 for all sites, representing typical periods in spring, summer, autumn, and winter, respectively.

**Evaluation of the simulated inorganic aerosol components.** For EC, NO_3_^-^, SO_4_^2-^, and NH_4_^+^, the simulation results of CMAQ/2D-VBS and the default CMAQv5.0.1 are consistent with each other (Supplementary Table 4-5, Supplementary Fig. 4-7). They both agree fairly well with the observations, with NO_3_^-^ somewhat overestimated and SO_4_^2-^ slightly underestimated; this is comparable with previous studies over East Asia[^23-25^](#_ENREF_23).

**Bias of the simulated fraction of OA consisting of SOA at the Jiaxing site (winter).** The fraction of OA consisting of SOA simulated by the Medium-Yield VBS and the High-Yield VBS agrees well with observations in all sites and periods except for overestimation at the Jiaxing site in winter. Here the fraction of OA consisting of SOA estimated with the PMF method and the OC/EC method are 31% and 55%, both lower than the simulated value of 72%-80%. Several reasons may account for this overestimation. First, the definition of SOA in CMAQ/2D-VBS differs from that in observations. In CMAQ/2D-VBS, POA is transformed to SOA once it undergoes a single step of oxidation. The PMF method splits OA into HOA, BBOA (both treated as POA), and OOA (treated as SOA) based on their similarity to POA and aged SOA in the atmosphere. For some large molecules in primary emissions, after a single step of oxidation, the majority of the molecule may still resemble POA and thus this mildly oxygenated material may be assigned into HOA/BBOA. As for the OC/EC method, an inherent assumption is that POA is nonvolatile and nonreactive. In fact, POA could evaporate, oxidize, and re-condense to form SOA. Therefore, the fraction of OA consisting of SOA derived from both the PMF method and the OC/EC method would be lower than simulations. In other words, it is rational if the simulated SOA fraction is slightly higher than observations. This reasoning also applies to other sites. Figure 2 shows that the High-Yield VBS also slightly overestimates the observed fraction of OA consisting of SOA in other sites. The results of High-Yield VBS would probably agree better with observations considering the description above. Second, the model somewhat overestimates the O_3_ concentrations for a few sites in winter[^26^](#_ENREF_26). We do not have O_3_ observations at Jiaxing site. If the O_3_ overestimation also occurs at Jiaxing site in winter, the SOA fraction will be overestimated as a consequence. Third, the smog-chamber experiments based on which the 2D-VBS parameterization were determined were all conducted at room temperature. The chemical mechanisms may be different at much lower temperature in winter, resulting in the model-measurement discrepancy in winter. The reasons for the underestimation in the fraction of OA consisting of SOA at Jiaxing site in winter need further exploration in future studies.

**Bias of the simulated O:C at the Changdao site.** The O:C simulated by the Medium-Yield VBS and the High-Yield VBS agrees well with observations for all sites and periods except that the aerosol O:C is underestimated by about 40% at the Changdao site. Hu et al.[^20^](#_ENREF_20) indicates that the observed O:C of HOA at the Changdao site is as high as 0.34, much higher than both previous observations (0.04–0.16) and the assumption of this study (Supplementary Table 3); this may be an important reason underlying the underestimation in O:C. On one hand, the observations at Changdao may be quite uncertain; on the other hand, the observations may imply that some special emission sources exist in the Changdao island. Besides, as an island in Bohai Bay dominated by aged aerosol, the underestimation of O:C in Changdao implies that the model may be still too conservative in the assumption of aging chemistry. Future studies are still needed to fully explain the bias at the Changdao site.

**Supplementary Table 4.** Comparison of simulated inorganic aerosol components with HR-ToF-AMS observations. The numbers in brackets represent NMBs.

|  | Observation | CMAQv5.0.1 | Medium-Yield VBS^a^ | High-Yield VBS^a^ |
| --- | --- | --- | --- | --- |
| **Pudong, Shanghai** | | | | |
| EC (μg m^-3^) | 2.0 | 2.8 (44%) | 2.8 (44%) | 2.8 (44%) |
| NO_3_^-^ (μg m^-3^) | 4.8 | 5.6 (17%) | 5.6 (18%) | 5.7 (19%) |
| SO_4_^2-^ (μg m^-3^) | 9.8 | 6.1 (-37%) | 6.1 (-37%) | 6.1 (-37%) |
| NH_4_^+^ (μg m^-3^) | 3.9 | 3.9 (0%) | 3.9 (1%) | 4.0 (1%) |
| **Changdao** | | | | |
| EC (μg m^-3^) | 2.5 | 3.9 (55%) | 3.9 (54%) | 3.9 (54%) |
| NO_3_^-^ (μg m^-3^) | 12.2 | 13.0 (7%) | 13.1 (8%) | 13.2 (8%) |
| SO_4_^2-^ (μg m^-3^) | 8.3 | 5.4 (-35%) | 5.5 (-34%) | 5.5 (-34%) |
| NH_4_^+^ (μg m^-3^) | 6.5 | 5.9 (-10%) | 5.9 (-9%) | 5.9 (-9%) |
| **Jiaxing (summer)** | | | | |
| EC (μg m^-3^) | 3.0 | 2.7 (-10%) | 2.7 (-9%) | 2.7 (-9%) |
| NO_3_^-^ (μg m^-3^) | 5.9 | 6.8 (16%) | 6.9 (17%) | 6.9 (17%) |
| SO_4_^2-^ (μg m^-3^) | 8.3 | 9.1 (10%) | 9.2 (10%) | 9.2 (10%) |
| NH_4_^+^ (μg m^-3^) | 4.2 | 5.4 (30%) | 5.5 (31%) | 5.5 (32%) |
| **Jiaxing (winter)** | | | | |
| EC (μg m^-3^) | 7.1 | 5.3 (-26%) | 5.2 (-27%) | 5.2 (-27%) |
| NO_3_^-^ (μg m^-3^) | 7.5 | 9.2 (23%) | 9.3 (24%) | 9.3 (25%) |
| SO_4_^2-^ (μg m^-3^) | 7.1 | 5.9 (-16%) | 6.0 (-15%) | 6.1 (-15%) |
| NH_4_^+^ (μg m^-3^) | 4.9 | 4.8 (-1%) | 4.9 (0%) | 4.9 (1%) |

a simulation results of the Low-Yield VBS configuration are not shown because it results in quite low OA concentrations and thus deviate significantly from observations.

|  |
| --- |

**Supplementary Figure 4.** Comparison of the simulated and observed concentrations of PM2.5 and it chemical components in Pudong, Shanghai. (**a**) PM2.5; (**b**) OC; (**c**) EC; (**d**) NO3-; (**e**) SO42-; (**f**) NH4+

|  |
| --- |

**Supplementary Figure 5.** Comparison of the simulated and observed concentrations of PM2.5 and it chemical components in SAES, Shanghai. (**a**) PM2.5; (**b**) OC; (**c**) EC; (**d**) NO3-; (**e**) SO42-; (**f**) NH4+

|  |
| --- |

**Supplementary Figure 6.** Comparison of the simulated and observed concentrations of PM2.5 and it chemical components in Nanjing. (**a**) PM2.5; (**b**) OC; (**c**) EC; (**d**) NO3-; (**e**) SO42-; (**f**) NH4+

|  |
| --- |

**Supplementary Figure 7.** Comparison of the simulated and observed concentrations of PM_2.5_ and it chemical components in Suzhou. (**a**) PM_2.5_; (**b**) OC; (**c**) EC; (**d**) NO_3_^-^; (**e**) SO_4_^2-^; (**f**) NH_4_^+^

**Supplementary Table 5.** Comparison of simulated and observed concentrations of PM_2.5_ and its chemical components at four long-term observational sites. The numbers in the table are NMBs.

| Time periods | PM_2.5_ | | | | | | OC | | | | | | EC | | NO_3_^-^ | | SO_4_^2-^ | | NH_4_^+^ | |
| --- | --- | --- | --- | --- | --- | --- | --- | --- | --- | --- | --- | --- | --- | --- | --- | --- | --- | --- | --- | --- |
|  | CMAQ^a^ | | M-VBS^a^ | | H-VBS^a^ | | CMAQ | | M-VBS | | H-VBS | | CMAQ | | CMAQ | | CMAQ | | CMAQ | |
| **Pudong, Shanghai** | | | | | | | | | | | | | | | | | | | | |
| 5.20–6.30 | | -18 | | -15 | | -11 | | -59 | | -52 | | -38 | | 16 | | 10 | | 8 | | -30 |
| 5.31–6.4^b^ | | -32 | | -26 | | -21 | | -74 | | -56 | | -42 | | - | | - | | - | | - |
| 7.20–8.20 | | 13 | | 19 | | 24 | | -36 | | -18 | | 0 | | 57 | | 98 | | -20 | | 32 |
| 11.7–11.30 | | -14 | | -12 | | -8 | | -53 | | -50 | | -33 | | 14 | | 26 | | -35 | | -36 |
| 11.11–11.15^b^ | | -29 | | -23 | | -17 | | -66 | | -44 | | -24 | | - | | - | | - | | - |
| 12.20–12.31 | | 12 | | 12 | | 19 | | -34 | | -39 | | -16 | | 33 | | 44 | | -37 | | -33 |
| 12.25–12.27^b^ | | 11 | | 14 | | 24 | | -38 | | -31 | | -4 | | - | | - | | - | | - |
| Average | | -2 | | 1 | | 6 | | -45 | | -40 | | -22 | | 30 | | 45 | | -21 | | -17 |
| **SAES, Shanghai** | | | | | | | | | | | | | | | | | | | | |
| 5.20–6.21 | -57 | | -56 | | -54 | | -39 | | -39 | | -21 | | 54 | | -9 | | 10 | | 3 | |
| 5.31–6.4 | -64 | | -60 | | -57 | | -80 | | -66 | | -55 | | - | | - | | - | | - | |
| 7.20–8.20 | -39 | | -35 | | -31 | | -46 | | -33 | | -17 | | 23 | | 40 | | -23 | | -17 | |
| 11.7–11.30 | -33 | | -32 | | -28 | | -42 | | -43 | | -25 | | 35 | | -2 | | -37 | | -11 | |
| 11.11–11.15 | -23 | | -15 | | -10 | | -56 | | -30 | | -7 | | - | | - | | - | | - | |
| 12.20–12.31 | -30 | | -31 | | -27 | | -27 | | -39 | | -19 | | 76 | | 3 | | -32 | | -11 | |
| 12.25–12.27 | -33 | | -31 | | -25 | | -31 | | -27 | | 1 | | - | | - | | - | | - | |
| Average | -40 | | -38 | | -35 | | -38 | | -39 | | -20 | | 47 | | 8 | | -20 | | -9 | |
| **Nanjing** | | | | | | | | | | | | | | | | | | | | |
| 5.20–6.30 | -32 | | -28 | | -24 | | -72 | | -63 | | -50 | | -35 | | 33 | | -19 | | -13 | |
| 5.31–6.4 | -50 | | -46 | | -42 | | -73 | | -62 | | -48 | | - | | - | | - | | - | |
| 7.20–8.20 | -17 | | -12 | | -7 | | -44 | | -28 | | -7 | | -5 | | 68 | | -26 | | 3 | |
| 11.7–11.30 | 2 | | 4 | | 10 | | -58 | | -57 | | -42 | | 0 | | 78 | | -38 | | -24 | |
| 11.11–11.15 | 10 | | 17 | | 26 | | -50 | | -31 | | -4 | | - | | - | | - | | - | |
| 12.20–12.31 | -18 | | -20 | | -15 | | -30 | | -44 | | -25 | | 7 | | 67 | | -35 | | -34 | |
| 12.25–12.27 | -40 | | -41 | | -37 | | -26 | | -37 | | -15 | | - | | - | | - | | - | |
| Average | -16 | | -14 | | -9 | | -51 | | -48 | | -31 | | -8 | | 61 | | -30 | | -17 | |
| **Suzhou** | | | | | | | | | | | | | | | | | | | | |
| 5.20–6.30 | -21 | | -17 | | -13 | | -51 | | -41 | | -24 | | 21 | | 10 | | -29 | | 21 | |
| 5.31–6.4 | -18 | | -10 | | -5 | | -67 | | -46 | | -29 | | - | | - | | - | | - | |
| 7.20–8.20 | 35 | | 50 | | 58 | | -29 | | 11 | | 38 | | 50 | | 119 | | -7 | | 121 | |
| 11.7–11.30 | -52 | | -50 | | -47 | | -77 | | -74 | | -64 | | -37 | | 9 | | -42 | | -50 | |
| 11.11–11.15 | -52 | | -46 | | -41 | | -80 | | -63 | | -48 | | - | | - | | - | | - | |
| 12.20–12.31 | -28 | | -27 | | -22 | | -82 | | -80 | | -72 | | -68 | | 40 | | -42 | | -44 | |
| 12.25–12.27 | -41 | | -38 | | -32 | | -75 | | -69 | | -56 | | - | | - | | - | | - | |
| Average | -17 | | -11 | | -6 | | -60 | | -46 | | -31 | | -8 | | 44 | | -30 | | 12 | |

^a^ “CMAQ” represents “the default CMAQv5.0.1”; “M-VBS” represents “the Medium-Yield VBS configuration”; “H-VBS” represents “the High-Yield VBS configuration”.

^b^ November 11–15, May 31–June 4, and December 25–27 are heavy-pollution episodes. Compared with the default CMAQv5.0.1, the Medium-Yield VBS and the High-Yield VBS show especially large improvement in model performance during these episodes.

**4. Effect of OA aging and intermediate-volatility emissions**

**Method to quantify the contributions of individual precursor class.** The default CMAQv5.0.1 does not include IVOC emissions, and POA is assumed to be nonvolatile and nonreactive. Therefore, IVOCs and POA do not contribute to SOA concentrations. The contributions of AVOCs and BVOCs to OA/SOA concentrations can be directly extracted from the model outputs. As for CMAQ/2D-VBS, the contribution of AVOCs and BVOCs to OA/SOA can be readily extracted because they are tracked separately in parallel layers of 2D-VBS. The contributions of POA and IVOCs are estimated with the “brute force” method. In detail, we run the model with POA or IVOC emissions turned off, and the differences between the simulated OA/SOA concentrations and the baseline OA/SOA concentrations represent the contribution of POA or IVOC emissions to OA/SOA concentrations. Note that the contributions of IVOCs to OA and SOA concentrations are theoretically identical. However, they are slightly different in Table 2 and Supplementary Table 7-8, attributable to the shift in gas-particle partitioning when the IVOC emissions are turned off.

**Seasonal and regional variations in the contributions of individual precursor classes.** The contributions of individual precursor classes differ to some extent in different seasons and regions. In spring/summer and over southern China, the contribution of NMVOCs, especially BVOCs, to OA concentration is higher than the average value, with that of POA lower than average. It is the other way around in autumn/winter and over northern China. This pattern is largely accounted for by the spatiotemporal distribution of primary emissions. The NMVOC emissions originating from solvent use are relatively higher and those from biogenic sources dramatically higher in spring/summer and over southern China, while the POA/IVOC emissions are relatively higher in autumn/winter and over northern China owing to intensive heating supply. In spite of the spatiotemporal variation, for most seasons and regions, IVOCs and POA are the two largest contributors to OA concentrations, and IVOCs stand out as the largest contributor to SOA concentrations.

|  | CMAQv5.0.1 | High-Yield VBS |
| --- | --- | --- |
| Jan | 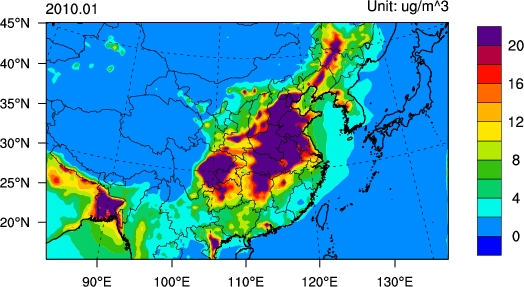 | 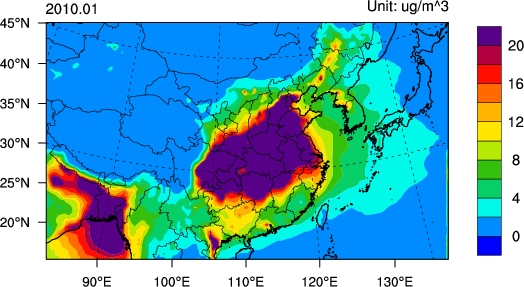 |
| May | 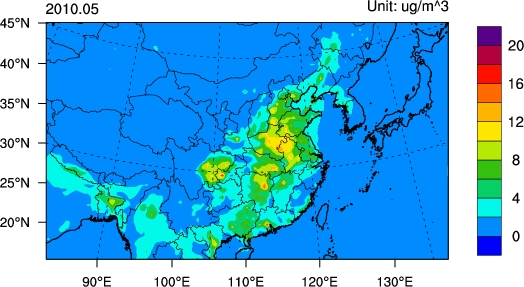 | 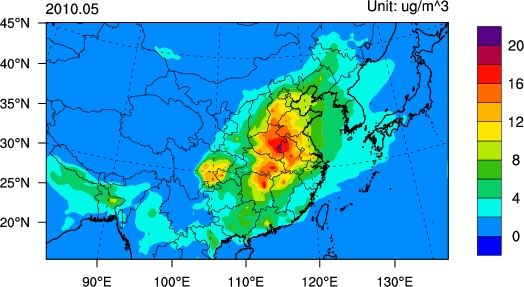 |
| Aug | 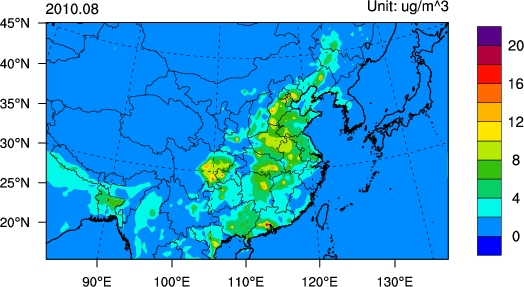 | 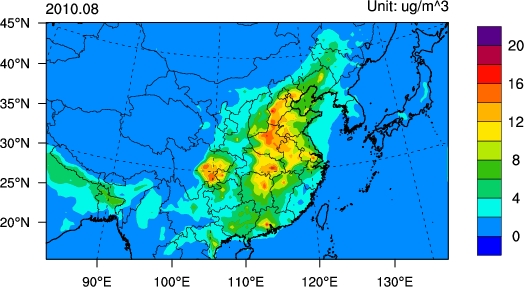 |
| Nov | 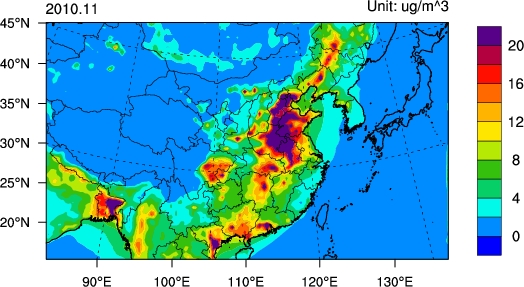 | 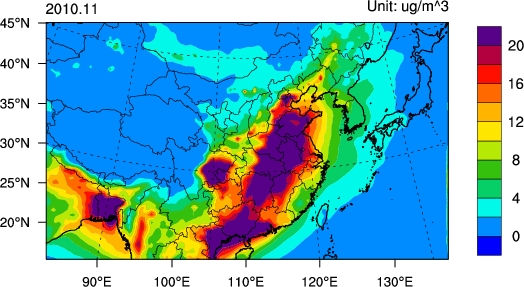 |

**Supplementary Figure 8.** Spatial distribution of simulated OA concentrations in January, May, August, and November, 2010. This figure is produced using the NCAR Command Language (Version 6.2.1) [Software]. (2014). Boulder, Colorado: UCAR/NCAR/CISL/TDD. http://dx.doi.org/10.5065/D6WD3XH5.

|  | CMAQv5.0.1 | High-Yield VBS |
| --- | --- | --- |
| Jan | 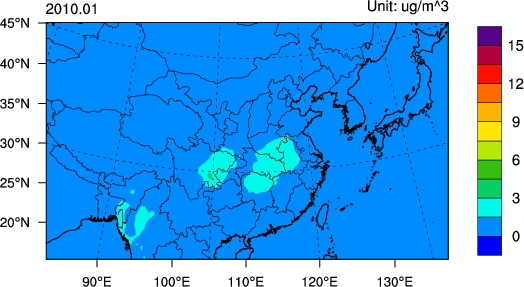 | 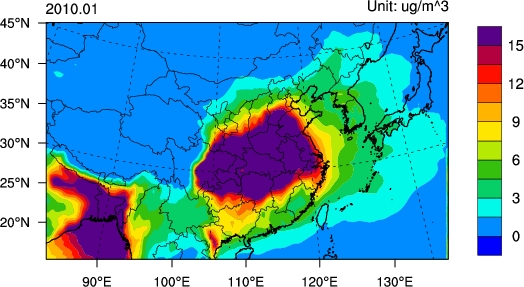 |
| May | 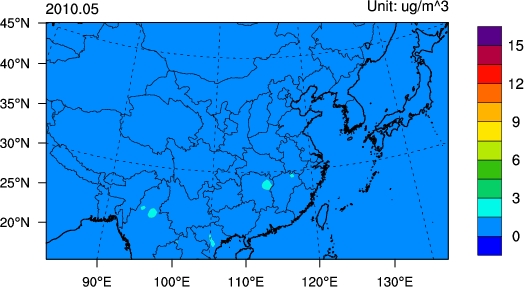 | 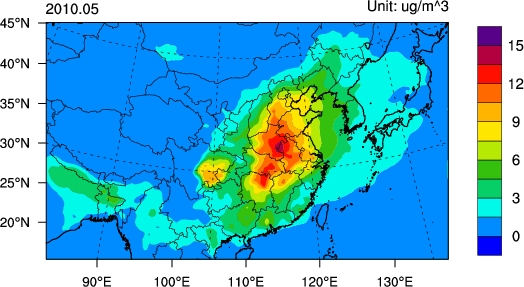 |
| Aug | 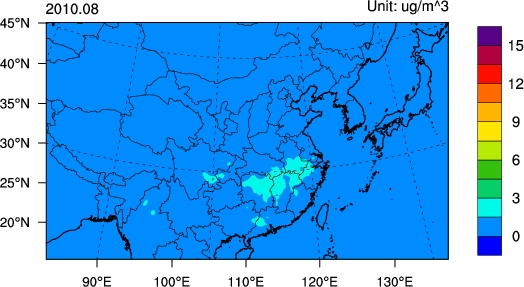 | 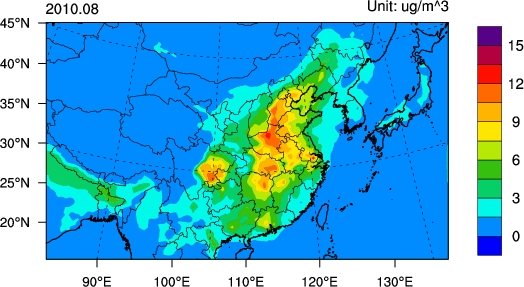 |
| Nov | 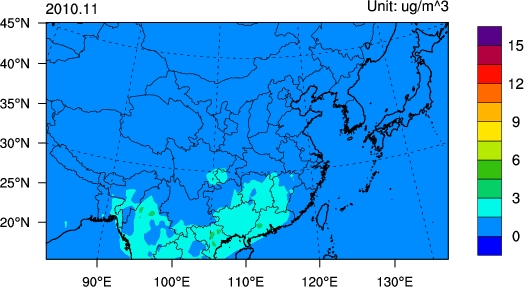 | 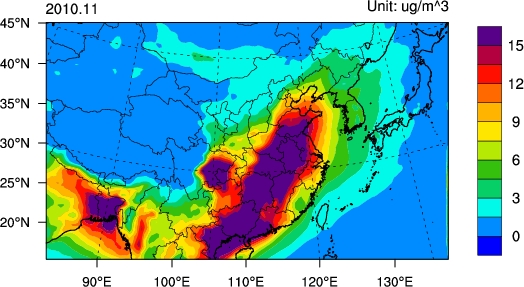 |

**Supplementary Figure 9.** Spatial distribution of simulated SOA concentrations in January, May, August, and November, 2010. This figure is produced using the NCAR Command Language (Version 6.2.1) [Software]. (2014). Boulder, Colorado: UCAR/NCAR/CISL/TDD. http://dx.doi.org/10.5065/D6WD3XH5.

**Supplementary Table 6.** The simulated OA and SOA concentrations (μg m^-3^), and O:C over key regions.

|  | Eastern China | | North China Plain | | Yangtze River Delta | | Pearl River Delta | | Sichuan Basin | |
| --- | --- | --- | --- | --- | --- | --- | --- | --- | --- | --- |
|  | CMAQ^a^ | H-VBS^a^ | CMAQ | H-VBS | CMAQ | H-VBS | CMAQ | H-VBS | CMAQ | H-VBS |
| **January** | | | | | | | | | | |
| OA | 14.0 | 18.0 | 22.3 | 21.0 | 13.0 | 20.5 | 7.2 | 7.9 | 27.5 | 38.4 |
| SOA | 0.9 | 12.9 | 0.8 | 12.2 | 1.1 | 16.2 | 0.8 | 5.7 | 1.8 | 27.9 |
| O:C | - | 0.31 | - | 0.26 | - | 0.33 | - | 0.33 | - | 0.34 |
| **May** | | | | | | | | | | |
| OA | 4.1 | 6.8 | 5.5 | 9.4 | 4.5 | 8.6 | 4.5 | 5.4 | 5.7 | 7.6 |
| SOA | 0.6 | 5.6 | 0.5 | 7.7 | 0.8 | 7.5 | 0.8 | 4.1 | 0.7 | 5.9 |
| O:C | - | 0.38 | - | 0.37 | - | 0.39 | - | 0.35 | - | 0.38 |
| **August** | | | | | | | | | | |
| OA | 3.9 | 6.1 | 5.3 | 8.6 | 3.9 | 7.1 | 5.9 | 7.2 | 5.7 | 7.8 |
| SOA | 0.9 | 5.1 | 0.7 | 7.0 | 1.3 | 6.2 | 1.2 | 5.6 | 1.0 | 6.2 |
| O:C | - | 0.38 | - | 0.37 | - | 0.38 | - | 0.35 | - | 0.39 |
| **November** | | | | | | | | | | |
| OA | 9.5 | 13.9 | 14.1 | 13.6 | 8.7 | 16.5 | 11.8 | 18.8 | 9.9 | 16.0 |
| SOA | 0.9 | 10.8 | 0.4 | 8.4 | 1.0 | 13.8 | 2.2 | 15.2 | 1.1 | 12.9 |
| O:C | - | 0.34 | - | 0.27 | - | 0.36 | - | 0.38 | - | 0.38 |
| **Four-month average** | | | | | | | | | | |
| OA | 7.9 | 11.2 | 11.8 | 13.2 | 7.5 | 13.2 | 7.3 | 9.8 | 12.2 | 17.5 |
| SOA | 0.8 | 8.6 | 0.6 | 8.8 | 1.1 | 10.9 | 1.2 | 7.6 | 1.1 | 13.2 |
| O:C | - | 0.35 | - | 0.32 | - | 0.36 | - | 0.35 | - | 0.37 |

^a^ “CMAQ” represents “the default CMAQv5.0.1”; “H-VBS” represents “the High-Yield VBS configuration”.

| Jan | May |
| --- | --- |
| 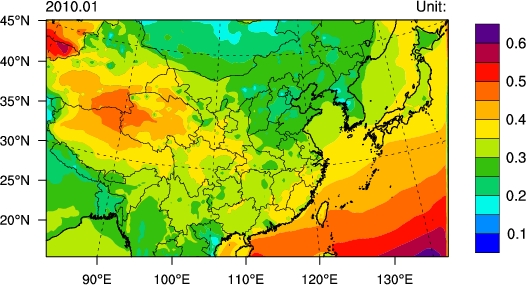 | 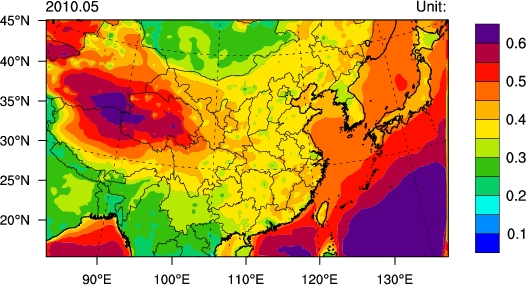 |
| Aug | Nov |
| 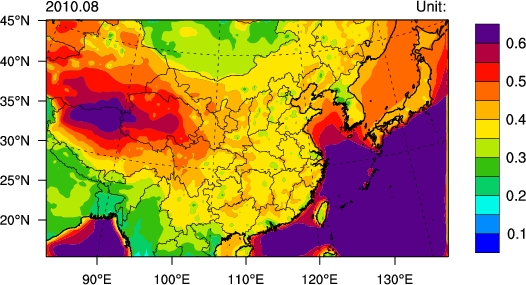 | 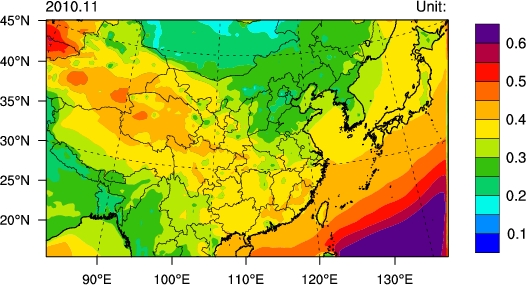 |

**Supplementary Figure 10.** Spatial distribution of simulated aerosol O:C by the High-Yield VBS configuration in January, May, August, and November, 2010. This figure is produced using the NCAR Command Language (Version 6.2.1) [Software]. (2014). Boulder, Colorado: UCAR/NCAR/CISL/TDD. http://dx.doi.org/10.5065/D6WD3XH5.

**Supplementary Table 7.** Contribution of individual precursor class to OA concentrations (μg m^-3^).

| Precursor class | Eastern China | | North China Plain | | Yangtze River Delta | | Pearl River Delta | | Sichuan Basin | |
| --- | --- | --- | --- | --- | --- | --- | --- | --- | --- | --- |
|  | CMAQ^a^ | H-VBS^a^ | CMAQ | H-VBS | CMAQ | H-VBS | CMAQ | H-VBS | CMAQ | H-VBS |
| **January** | | | | | | | | | | |
| AVOCs | 0.6 | 1.5 | 0.5 | 1.2 | 0.9 | 2.6 | 0.4 | 1.1 | 1.3 | 2.8 |
| BVOCs | 0.3 | 0.2 | 0.2 | 0.1 | 0.3 | 0.2 | 0.3 | 0.4 | 0.5 | 0.4 |
| POA | 13.1 | 8.7 | 21.6 | 12.9 | 11.9 | 8.5 | 6.4 | 3.6 | 25.7 | 18.4 |
| IVOCs | - | 8.5 | - | 8.3 | - | 10.5 | - | 3.3 | - | 18.3 |
| **May** | | | | | | | | | | |
| AVOCs | 0.2 | 0.7 | 0.3 | 1.0 | 0.4 | 1.4 | 0.3 | 0.8 | 0.2 | 0.6 |
| BVOCs | 0.3 | 0.6 | 0.2 | 0.3 | 0.4 | 0.7 | 0.5 | 0.8 | 0.4 | 0.7 |
| POA | 3.5 | 2.4 | 5.1 | 3.5 | 3.7 | 2.6 | 3.7 | 2.1 | 5.0 | 3.2 |
| IVOCs | - | 3.3 | - | 5.0 | - | 4.4 | - | 2.1 | - | 3.4 |
| **August** | | | | | | | | | | |
| AVOCs | 0.2 | 0.6 | 0.3 | 1.0 | 0.3 | 1.0 | 0.3 | 1.0 | 0.3 | 0.6 |
| BVOCs | 0.6 | 1.1 | 0.3 | 0.6 | 1.0 | 2.0 | 0.9 | 1.5 | 0.7 | 1.3 |
| POA | 3.0 | 2.0 | 4.7 | 3.1 | 2.6 | 1.7 | 4.7 | 2.6 | 4.7 | 3.1 |
| IVOCs | - | 2.6 | - | 4.3 | - | 2.7 | - | 2.6 | - | 3.3 |
| **November** | | | | | | | | | | |
| AVOCs | 0.4 | 1.2 | 0.3 | 0.6 | 0.6 | 2.2 | 0.8 | 2.4 | 0.6 | 1.4 |
| BVOCs | 0.5 | 0.6 | 0.1 | 0.1 | 0.3 | 0.4 | 1.4 | 2.1 | 0.5 | 0.6 |
| POA | 8.6 | 5.8 | 13.7 | 8.0 | 7.7 | 5.8 | 9.6 | 6.7 | 8.8 | 6.4 |
| IVOCs | - | 7.0 | - | 5.9 | - | 9.0 | - | 8.6 | - | 8.1 |
| **Four-month average** | | | | | | | | | | |
| AVOCs | 0.4 | 1.0 | 0.4 | 1.0 | 0.5 | 1.8 | 0.4 | 1.3 | 0.6 | 1.4 |
| BVOCs | 0.4 | 0.6 | 0.2 | 0.2 | 0.5 | 0.8 | 0.8 | 1.2 | 0.5 | 0.8 |
| POA | 7.1 | 4.7 | 11.2 | 6.9 | 6.5 | 4.6 | 6.1 | 3.8 | 11.1 | 7.8 |
| IVOCs | - | 5.4 | - | 5.9 | - | 6.6 | - | 4.2 | - | 8.3 |

^a^ “CMAQ” represents “the default CMAQv5.0.1”; “H-VBS” represents “the High-Yield VBS configuration”.

**Supplementary Table 8.** Contribution of individual precursor class to SOA concentrations (μg m^-3^).

| Precursor class | Eastern China | | North China Plain | | Yangtze River Delta | | Pearl River Delta | | Sichuan Basin | |
| --- | --- | --- | --- | --- | --- | --- | --- | --- | --- | --- |
|  | CMAQ^a^ | H-VBS^a^ | CMAQ | H-VBS | CMAQ | H-VBS | CMAQ | H-VBS | CMAQ | H-VBS |
| **January** | | | | | | | | | | |
| AVOCs | 0.6 | 1.5 | 0.5 | 1.2 | 0.9 | 2.6 | 0.4 | 1.1 | 1.3 | 2.8 |
| BVOCs | 0.3 | 0.2 | 0.2 | 0.1 | 0.3 | 0.2 | 0.3 | 0.4 | 0.5 | 0.4 |
| POA | 0.0 | 3.6 | 0.0 | 4.0 | 0.0 | 4.1 | 0.0 | 1.4 | 0.0 | 7.8 |
| IVOCs | - | 8.1 | - | 7.8 | - | 10.1 | - | 3.2 | - | 17.4 |
| **May** | | | | | | | | | | |
| AVOCs | 0.2 | 0.7 | 0.3 | 1.0 | 0.4 | 1.4 | 0.3 | 0.8 | 0.2 | 0.6 |
| BVOCs | 0.3 | 0.6 | 0.2 | 0.3 | 0.4 | 0.7 | 0.5 | 0.8 | 0.4 | 0.7 |
| POA | 0.0 | 1.3 | 0.0 | 1.8 | 0.0 | 1.4 | 0.0 | 0.8 | 0.0 | 1.5 |
| IVOCs | - | 3.2 | - | 4.8 | - | 4.2 | - | 2.0 | - | 3.3 |
| **August** | | | | | | | | | | |
| AVOCs | 0.2 | 0.6 | 0.3 | 1.0 | 0.3 | 1.0 | 0.3 | 1.0 | 0.3 | 0.6 |
| BVOCs | 0.6 | 1.1 | 0.3 | 0.6 | 1.0 | 2.0 | 0.9 | 1.5 | 0.7 | 1.3 |
| POA | 0.0 | 1.0 | 0.0 | 1.5 | 0.0 | 0.8 | 0.0 | 1.0 | 0.0 | 1.5 |
| IVOCs | - | 2.5 | - | 4.2 | - | 2.7 | - | 2.5 | - | 3.1 |
| **November** | | | | | | | | | | |
| AVOCs | 0.4 | 1.2 | 0.3 | 0.6 | 0.6 | 2.2 | 0.8 | 2.4 | 0.6 | 1.4 |
| BVOCs | 0.5 | 0.6 | 0.1 | 0.1 | 0.3 | 0.4 | 1.4 | 2.1 | 0.5 | 0.6 |
| POA | 0.0 | 2.7 | 0.0 | 2.7 | 0.0 | 3.1 | 0.0 | 3.1 | 0.0 | 3.3 |
| IVOCs | - | 6.7 | - | 5.5 | - | 8.7 | - | 8.3 | - | 7.8 |
| **Four-month average** | | | | | | | | | | |
| AVOCs | 0.4 | 1.0 | 0.4 | 1.0 | 0.5 | 1.8 | 0.4 | 1.3 | 0.6 | 1.4 |
| BVOCs | 0.4 | 0.6 | 0.2 | 0.2 | 0.5 | 0.8 | 0.8 | 1.2 | 0.5 | 0.8 |
| POA | 0.0 | 2.2 | 0.0 | 2.5 | 0.0 | 2.4 | 0.0 | 1.6 | 0.0 | 3.5 |
| IVOCs | - | 5.1 | - | 5.6 | - | 6.4 | - | 4.0 | - | 7.9 |

^a^ “CMAQ” represents “the default CMAQv5.0.1”; “H-VBS” represents “the High-Yield VBS configuration”.

**5. Sensitivity analysis of key uncertain parameters**

**Design of the sensitivity scenarios.** We designed a series of sensitivity scenarios to assess the impact of key factors on simulated OA properties and effect of OA aging and intermediate-volatility emissions, and to explore the possible reasons for the discrepancy between model and measurements. As described in the main text, we evaluated the effect of OA aging and intermediate-volatility emissions primarily based on the results of the High-Yield VBS configuration because it agrees best with field observations. Here we also used the High-Yield VBS as the reference (i.e., base case) for sensitivity analysis. We conducted the sensitivity analysis during the periods when the HR-ToF-AMS observations are available to facilitate the comparison of the sensitivity runs with observations.

The definitions of the sensitivity scenarios designed in this study are summarized in Supplementary Table 9. The uncertainty of emission inventory is a crucial contributor to the uncertainty of simulation results. Integrating the results of previous studies, we estimated the 95% confidence interval of anthropogenic NMVOC emissions and primary OC emissions to be [-40%, +100%] and [-40%, +121%], respectively. The current emission inventory does not include emissions of IVOCs. In this study, we assumed that the IVOC emissions were proportional to POA emissions based on the measurements in the diluted emission experiments. Here we assumed the uncertainty range of POA/IVOC emissions to be the same of OC emissions (i.e., [-40%, +121%]). We note that the uncertainty in IVOC emissions could be even larger considering our poor understanding of IVOC emissions. On the basis of the information above, we designed a “high anthropogenic NMVOCs” scenario and a “low anthropogenic NMVOCs”, assuming the anthropogenic NMVOC emissions to be 2.0 times and 60% of the base-case emissions, respectively. Similarly, we designed a “high POA/IVOCs” scenario and a “low POA/IVOCs” scenario, with the assumptions that the POA/IVOC emissions account for 2.2 times and 60% of the base-case emissions, respectively. The uncertainty in the volatility distribution and the O:C ratio of POA also contributes to the uncertainty of the simulation results. In the base case, the volatility distribution of POA was consistent with the “median” measurements of May et al.[^33-35^](#_ENREF_33). May et al.[^33-35^](#_ENREF_33) also presented the uncertainty range defined by the 25^th^ percentile and 75^th^ percentile of the measurements. We developed a “high POA volatility” scenario and a “low POA volatility” scenario in which the mass fraction of high volatility bins and low volatility bins reach the upper bound of the uncertainty range, respectively. Note that the uncertainty of POA volatility may be even larger if the measurements of other studies are included. The volatility distributions of POA in these two sensitivity scenarios are illustrated in Supplementary Table 10. Besides, although previous studies demonstrated a robust inverse correlation between O:C and volatility, the quantitative relationship between O:C and C^*^ was not achievable based purely on measurements. To test the uncertainty in the O:C distribution, we developed a “O:C of POA fixed” scenario where the O:C of POA does not change with C^*^. We assigned the O:C of biomass burning emissions a fixed value of 0.20, and that of other sources was assigned 0.08. We have emphasized the large variability in the POA/IVOC oxidation chemistry, and designed three 2D-VBS configurations to account for this variability, including the High-Yield VBS (i.e., base case for sensitivity analysis), the Medium-Yield VBS, and the Low-Yield VBS. Here we also included the Medium-Yield VBS configuration as one sensitivity scenario. We eliminated the Low-Yield VBS because it deviated significantly from field observations and was not likely a plausible configuration. We further designed a Very-High-Yield VBS configuration with which the 10^th^ percentile of the simulated to measured OA was 1.0 for the diluted emission experiments.

**Supplementary Table 9.** Summary of the definitions of the sensitivity scenarios

| NO. | Scenario name | Parameter | Value in the base case | Value in the sensitivity scenario |
| --- | --- | --- | --- | --- |
| 1 | High anthropogenic NMVOCs | Anthropogenic NMVOC emissions | Base-case emissions | 2.0×base-case emissions |
| 2 | Low anthropogenic NMVOCs | Anthropogenic NMVOC emissions | Base-case emissions | 0.6×base-case emissions |
| 3 | High POA/IVOCs | POA/IVOC emissions | Base-case emissions | 2.2×base-case emissions |
| 4 | Low POA/IVOCs | POA/IVOC emissions | Base-case emissions | 0.6×base-case emissions |
| 5 | High POA volatility | The volatility distribution of POA emissions | Base-case volatility distribution | Mass fraction of high volatility bins reach the upper bound of the uncertainty range |
| 6 | Low POA volatility | The volatility distribution of POA emissions | Base-case volatility distribution | Mass fraction of low volatility bins reach the upper bound of the uncertainty range |
| 7 | O:C of POA fixed | The O:C distribution of POA/IVOC emissions | O:C decreases with the increase of C^*^ | O:C does not change with C^*^; the O:C of biomass burning emissions is assigned a fixed value of 0.20, and that of other sources 0.08 |
| 8 | Very-High-Yield VBS | 2D-VBS parameterization of photo-oxidation of POA/IVOCs | The parameterization for which the 25^th^ percentile of simulated to measured OA equals1.0 for the diluted emission experiments | The parameterization for which the 10^th^ percentile of simulated to measured OA equals1.0 for the diluted emission experiments |
| 9 | Medium-Yield VBS | 2D-VBS parameterization of photo-oxidation of POA/IVOCs | The parameterization for which the 25^th^ percentile of simulated to measured OA equals1.0 for the diluted emission experiments | The parameterization for which the 50^th^ percentile of simulated to measured OA equals1.0 for the diluted emission experiments |

**Supplementary Table 10.** The mass fraction of POA emissions in each volatility bin in the base-case simulation and sensitivity scenarios.

| log_10_C^*^ (μg m^-3^) | | -2 | 0 | 1 | 2 | 3 | 4 |
| --- | --- | --- | --- | --- | --- | --- | --- |
| Gasoline vehicles | Base case | 0.27 | 0.15 | 0.26 | 0.15 | 0.03 | 0.14 |
|  | High volatility | 0.16 | 0.11 | 0.21 | 0.19 | 0.08 | 0.25 |
|  | Low volatility | 0.34 | 0.21 | 0.30 | 0.10 | 0.02 | 0.03 |
| Diesel vehicles | Base case | 0.03 | 0.25 | 0.37 | 0.24 | 0.06 | 0.05 |
|  | High volatility | 0.02 | 0.12 | 0.39 | 0.26 | 0.08 | 0.13 |
|  | Low volatility | 0.11 | 0.29 | 0.36 | 0.17 | 0.04 | 0.03 |
| Biomass burning | Base case | 0.2 | 0.1 | 0.1 | 0.2 | 0.1 | 0.3 |
|  | High volatility | 0.15 | 0.05 | 0.05 | 0.2 | 0.15 | 0.4 |
|  | Low volatility | 0.25 | 0.15 | 0.15 | 0.2 | 0.05 | 0.2 |
| Other sources | Base case | 0.167 | 0.167 | 0.243 | 0.197 | 0.063 | 0.163 |
|  | High volatility | 0.110 | 0.093 | 0.217 | 0.217 | 0.103 | 0.260 |
|  | Low volatility | 0.234 | 0.217 | 0.270 | 0.157 | 0.036 | 0.086 |

**Supplementary Table 11.** The simulated OA concentrations (μg m^-3^), O:C, and the fraction of OA consisting of SOA (%) in the sensitivity scenarios.

|  | Observation | Base case | High anthropogenic NMVOCs | Low anthropogenic NMVOCs | High POA  /IVOCs | Low POA  /IVOCs | High POA volatility | Low POA volatility | O:C of POA fixed | Very-High-Yield VBS | Medium-Yield VBS |
| --- | --- | --- | --- | --- | --- | --- | --- | --- | --- | --- | --- |
| **Pudong, Shanghai** | | | | | | | | | | | |
| OA | 8.4 | 6.3 | 7.7 | 5.5 | 12.7 | 4.2 | 5.9 | 6.6 | 6.1 | 6.8 | 4.6 |
| O:C | 0.28 | 0.31 | 0.33 | 0.31 | 0.28 | 0.34 | 0.33 | 0.30 | 0.35 | 0.32 | 0.29 |
| SOA% | 76 | 79 | 83 | 77 | 74 | 83 | 85 | 74 | 79 | 81 | 73 |
| **Changdao** | | | | | | | | | | | |
| OA | 13.4 | 13.2 | 16.1 | 11.7 | 28.5 | 8.3 | 12.3 | 14.1 | 12.7 | 14.3 | 9.3 |
| O:C | 0.59 | 0.33 | 0.35 | 0.32 | 0.31 | 0.35 | 0.35 | 0.33 | 0.40 | 0.35 | 0.31 |
| SOA% | 70 | 75 | 79 | 72 | 71 | 77 | 82 | 68 | 74 | 76 | 66 |
| **Jiaxing (summer)** | | | | | | | | | | | |
| OA | 10.6 | 5.9 | 6.9 | 5.5 | 11.0 | 4.3 | 5.6 | 6.3 | 5.8 | 6.4 | 4.8 |
| O:C | 0.29 | 0.32 | 0.34 | 0.31 | 0.29 | 0.35 | 0.33 | 0.31 | 0.35 | 0.33 | 0.31 |
| SOA% | 68/64^a^ | 82 | 84 | 81 | 76 | 86 | 87 | 77 | 81 | 83 | 78 |
| **Jiaxing (winter)** | | | | | | | | | | | |
| OA | 12.8 | 14.7 | 20.5 | 11.6 | 31.8 | 9.1 | 13.9 | 15.3 | 13.9 | 16.0 | 10.3 |
| O:C | 0.33 | 0.30 | 0.32 | 0.28 | 0.28 | 0.31 | 0.31 | 0.29 | 0.35 | 0.31 | 0.28 |
| SOA% | 31/55^a^ | 80 | 85 | 75 | 77 | 82 | 85 | 74 | 79 | 81 | 72 |

^a^ the values on the left and right sides of the virgule represent the fraction of OA consisting of SOA estimated with the PMF method and the OC/EC method, respectively.

REFERENCES

1 Donahue, N. M. *et al.* Aging of biogenic secondary organic aerosol via gas-phase OH radical reactions. *P. Natl. Acad. Sci. USA.* **109**, 13503-13508, DOI 10.1073/pnas.1115186109 (2012).

2 Chen, S., Brune, W. H., Lambe, A. T., Davidovits, P. & Onasch, T. B. Modeling organic aerosol from the oxidation of alpha-pinene in a Potential Aerosol Mass (PAM) chamber. *Atmos. Chem. Phys.* **13**, 5017-5031, DOI 10.5194/acp-13-5017-2013 (2013).

3 Chacon-Madrid, H. J., Murphy, B. N., Pandis, S. N. & Donahue, N. M. Simulations of Smog-Chamber Experiments Using the Two-Dimensional Volatility Basis Set: Linear Oxygenated Precursors. *Environ. Sci. Technol.* **46**, 11179-11186, DOI 10.1021/Es3017232 (2012).

4 Murphy, B. N., Donahue, N. M., Fountoukis, C. & Pandis, S. N. Simulating the oxygen content of ambient organic aerosol with the 2D volatility basis set. *Atmos. Chem. Phys.* **11**, 7859-7873, DOI 10.5194/acp-11-7859-2011 (2011).

5 Murphy, B. N. *et al.* Functionalization and fragmentation during ambient organic aerosol aging: application of the 2-D volatility basis set to field studies. *Atmos. Chem. Phys.* **12**, 10797-10816, DOI 10.5194/acp-12-10797-2012 (2012).

6 Roldin, P. *et al.* Development and evaluation of the aerosol dynamics and gas phase chemistry model ADCHEM. *Atmos. Chem. Phys.* **11**, 5867-5896, DOI 10.5194/acp-11-5867-2011 (2011).

7 Zhao, B. *et al.* Evaluation of one-dimensional and two-dimensional volatility basis sets in simulating the aging of secondary organic aerosols with smog-chamber experiments. *Environ. Sci. Technol.* **49**, 2245-2254, DOI 10.1021/es5048914 (2015).

8 Carlton, A. G. *et al.* Model Representation of Secondary Organic Aerosol in CMAQv4.7. *Environ. Sci. Technol.* **44**, 8553-8560, DOI 10.1021/Es100636q (2010).

9 Ziemann, P. J. & Atkinson, R. Kinetics, products, and mechanisms of secondary organic aerosol formation. *Chem. Soc. Rev.* **41**, 6582-6605, DOI 10.1039/C2cs35122f (2012).

10 Pankow, J. F. & Asher, W. E. SIMPOL.1: a simple group contribution method for predicting vapor pressures and enthalpies of vaporization of multifunctional organic compounds. *Atmos. Chem. Phys.* **8**, 2773-2796 (2008).

11 Zhao, B. *et al.* Impact of national NOx and SO2 control policies on particulate matter pollution in China. *Atmos. Environ.* **77**, 453-463, DOI 10.1016/j.atmosenv.2013.05.012 (2013).

12 Zhao, B. *et al.* Environmental effects of the recent emission changes in China: implications for particulate matter pollution and soil acidification. *Environ. Res. Lett.* **8**, 024031, DOI 10.1088/1748-9326/8/2/024031 (2013).

13 Wang, S. X. *et al.* Emission trends and mitigation options for air pollutants in East Asia. *Atmos. Chem. Phys.* **14**, 6571-6603, DOI 10.5194/acp-14-6571-2014 (2014).

14 Fu, X. *et al.* Estimating NH3 emissions from agricultural fertilizer application in China using the bi-directional CMAQ model coupled to an agro-ecosystem model. *Atmos. Chem. Phys.* **15**, 6637-6649, DOI 10.5194/acp-15-6637-2015 (2015).

15 Zhang, Q. *et al.* Asian emissions in 2006 for the NASA INTEX-B mission. *Atmos. Chem. Phys.* **9**, 5131-5153 (2009).

16 Fu, X. *et al.* Emission inventory of primary pollutants and chemical speciation in 2010 for the Yangtze River Delta region, China. *Atmos. Environ.* **70**, 39-50, DOI 10.1016/j.atmosenv.2012.12.034 (2013).

17 Guenther, A. *et al.* Estimates of global terrestrial isoprene emissions using MEGAN (Model of Emissions of Gases and Aerosols from Nature). *Atmos. Chem. Phys.* **6**, 3181-3210 (2006).

18 Huang, X. F. *et al.* Highly time-resolved chemical characterization of atmospheric fine particles during 2010 Shanghai World Expo. *Atmos. Chem. Phys.* **12**, 4897-4907, DOI 10.5194/acp-12-4897-2012 (2012).

19 Huang, X. F. *et al.* Highly time-resolved carbonaceous aerosol characterization in Yangtze River Delta of China: Composition, mixing state and secondary formation. *Atmos. Environ.* **64**, 200-207, DOI 10.1016/j.atmosenv.2012.09.059 (2013).

20 Hu, W. W. *et al.* Insights on organic aerosol aging and the influence of coal combustion at a regional receptor site of central eastern China. *Atmos. Chem. Phys.* **13**, 10095-10112, DOI 10.5194/acp-13-10095-2013 (2013).

21 Cheng, Z. *et al.* Estimation of Aerosol Mass Scattering Efficiencies under High Mass Loading: Case Study for the Megacity of Shanghai, China. *Environ. Sci. Technol.* **49**, 831-838, Doi 10.1021/Es504567q (2015).

22 Cheng, Z. *et al.* Impact of biomass burning on haze pollution in the Yangtze River delta, China: a case study in summer 2011. *Atmos. Chem. Phys.* **14**, 4573-4585, DOI 10.5194/acp-14-4573-2014 (2014).

23 Wang, S. X. *et al.* Verification of anthropogenic emissions of China by satellite and ground observations. *Atmos. Environ.* **45**, 6347-6358, DOI 10.1016/j.atmosenv.2011.08.054 (2011).

24 Gao, Y., Zhao, C., Liu, X. H., Zhang, M. G. & Leung, L. R. WRF-Chem simulations of aerosols and anthropogenic aerosol radiative forcing in East Asia. *Atmos. Environ.* **92**, 250-266, DOI 10.1016/j.atmosenv.2014.04.038 (2014).

25 Wang, Y., Zhang, Q. Q., He, K., Zhang, Q. & Chai, L. Sulfate-nitrate-ammonium aerosols over China: response to 2000-2015 emission changes of sulfur dioxide, nitrogen oxides, and ammonia. *Atmos. Chem. Phys.* **13**, 2635-2652, DOI 10.5194/acp-13-2635-2013 (2013).

26 Zhao, B. *Numerical Simulation of the Chemical Components of Fine Particles and their Response to Precursor Emissions* Doctor thesis, Tsinghua University, (2015).

27 Jimenez, J. L. *et al.* Evolution of Organic Aerosols in the Atmosphere. *Science* **326**, 1525-1529, DOI 10.1126/science.1180353 (2009).

28 Ng, N. L. *et al.* Organic aerosol components observed in Northern Hemispheric datasets from Aerosol Mass Spectrometry. *Atmos. Chem. Phys.* **10**, 4625-4641, DOI 10.5194/acp-10-4625-2010 (2010).

29 Gong, Z. H. *et al.* Characterization of submicron aerosols in the urban outflow of the central Pearl River Delta region of China. *Front. Env. Sci. Eng.* **6**, 725-733, DOI 10.1007/s11783-012-0441-8 (2012).

30 Wei, W. *et al.* Emission and speciation of non-methane volatile organic compounds from anthropogenic sources in China. *Atmos. Environ.* **42**, 4976–4988 (2008).

31 Bo, Y., Cai, H. & Xie, S. D. Spatial and temporal variation of historical anthropogenic NMVOCs emission inventories in China. *Atmos. Chem. Phys.* **8**, 7297–7316 (2008).

32 Zhao, Y., Nielsen, C. P., Lei, Y., McElroy, M. B. & Hao, J. Quantifying the uncertainties of a bottom-up emission inventory of anthropogenic atmospheric pollutants in China. *Atmos. Chem. Phys.* **11**, 2295-2308, DOI 10.5194/acp-11-2295-2011 (2011).

33 May, A. A. *et al.* Gas-Particle Partitioning of Primary Organic Aerosol Emissions: (2) Diesel Vehicles. *Environ. Sci. Technol.* **47**, 8288-8296, Doi 10.1021/Es400782j (2013).

34 May, A. A. *et al.* Gas-particle partitioning of primary organic aerosol emissions: (1) Gasoline vehicle exhaust. *Atmos. Environ.* **77**, 128-139, DOI 10.1016/j.atmosenv.2013.04.060 (2013).

35 May, A. A. *et al.* Gas-particle partitioning of primary organic aerosol emissions: 3. Biomass burning. *J. Geophys. Res-Atmos.* **118**, 11327-11338, Doi 10.1002/Jgrd.50828 (2013).

36 Huffman, J. A. *et al.* Chemically-Resolved Volatility Measurements of Organic Aerosol from Different Sources. *Environ. Sci. Technol.* **43**, 5351-5357, Doi 10.1021/Es803539d (2009).

37 Chen, Q., Liu, Y. J., Donahue, N. M., Shilling, J. E. & Martin, S. T. Particle-Phase Chemistry of Secondary Organic Material: Modeled Compared to Measured O:C and H:C Elemental Ratios Provide Constraints. *Environ. Sci. Technol.* **45**, 4763-4770, Doi 10.1021/Es104398s (2011).
